# Genetic variation for rectal gland volatiles among recently collected isofemale lines and a domesticated strain of Queensland fruit fly, *Bactrocera tryoni* (Diptera: Tephritidae)

**DOI:** 10.1371/journal.pone.0285099

**Published:** 2023-04-28

**Authors:** Cynthia Castro-Vargas, Gunjan Pandey, Heng Lin Yeap, Shirleen S. Prasad, Michael J. Lacey, Siu Fai Lee, Soo J. Park, Phillip W. Taylor, John G. Oakeshott

**Affiliations:** 1 Environment, Black Mountain, Commonwealth Scientific and Industrial Research Organisation, Acton, ACT, Australia; 2 Applied BioSciences, Macquarie University, North Ryde, NSW, Australia; 3 Australian Research Council Centre for Fruit Fly Biosecurity Innovation, Macquarie University, North Ryde, NSW, Australia; 4 Bio21 Molecular Science and Biotechnology Institute, University of Melbourne, Parkville, VIC, Australia; 5 National Collections and Marine Infrastructure, Black Mountain, Commonwealth Scientific and Industrial Research Organisation, Acton, ACT, Australia; Albert-Ludwigs-Universitat Freiburg, GERMANY

## Abstract

Divergence between populations in mating behaviour can function as a potent premating isolating mechanism and promote speciation. However, very few cases of inherited intraspecific variation in sexual signalling have been reported in tephritid fruit flies, despite them being a highly speciose family. We tested for such variation in one tephritid, the Queensland fruit fly, *Bactrocera tryoni* (Qfly). Qfly mating behaviour depends on volatiles secreted from male rectal glands but no role for the volatiles from female rectal glands has yet been reported. We previously detected over 100 volatile compounds in male rectal glands and identified over 30 of them. Similar numbers were recorded in females. However, many compounds showed presence/absence differences between the sexes and many others showed quantitative differences between them. Here we report inherited variation among 24 Qfly lines (23 isofemale lines established from recent field collections and one domesticated line) in the abundance of three esters, two alcohols, two amides, an aldehyde and 18 unidentified volatiles in male rectal glands. We did not find any compounds in female rectal glands that varied significantly among the lines, although this may at least partly reflect lower female sample numbers. Most of the 26 male compounds that differed between lines were more abundant in the domesticated line than any of the recently established isofemale lines, which concurs with other evidence for changes in mating behaviour during domestication of this species. There were also large differences in several of the 26 compounds among the isofemale lines, and some of these differences were associated with the regions from which the lines were collected. While some of the variation in different compounds was correlated across lines, much of it was not, implicating involvement of multiple genes. Our findings parallel reports of geographic variation in other Qfly traits and point to inherited differences in reproductive physiology that could provide a basis for evolution of premating isolation between ecotypes.

## Introduction

Pheromones involved in mating behaviour have been found to function as effective premating isolating mechanisms among sympatric populations of many closely related insect species; in some cases quite subtle differences in pheromone composition can be sufficient to deter interspecific mating [1–4]. However, much less is known about intraspecific variation in pheromone composition, and less still about its genetic basis. Two major exceptions involve moths; in one case racial differences in pheromone content in *Ostrinia nubilalis* were largely attributed to allelic differences at a single locus affecting a fatty acyl reductase activity [5], whereas in the other case quantitative intra-population variation in an aldehyde component of a *Heliothis virescens* pheromone was found to be polygenically inherited, with the major locus only explaining 11–21% of the variation [6]. Although the genetic architectures were very different, both cases exemplify a situation where genetic polymorphism in pheromone composition within a species might potentially provide a basis for the development of a premating isolating mechanism. Variations in sex pheromone composition between geographic populations of other moths [7–10] and a sandfly [11, 12] have also been reported but the genetic bases of these differences are not yet known.

Tephritid fruit flies are a promising model to further investigate intraspecific polymorphism in pheromone composition and its potential to drive non-random mating. Several of the key genera are highly speciose [13, 14], and some now-sympatric sibling species pairs have been shown to be effectively isolated by differences in mating behaviours [15, 16]. Moreover many are important horticultural pests [17, 18], and there has been considerable research into biologically based control methods such as the Sterile Insect Technique (SIT) and Male Annihilation Technique (MAT) which exploit and/or intervene in aspects of mating or more general sex-specific behaviours [19–25]. There is also increasing interest in the biochemistries underpinning those behaviours, which are often found to involve semiochemicals [26–34]. Male rectal glands have been shown to produce many of these compounds in various tephritids, including the Mexican fruit fly, *Anastrepha ludens* [35, 36], Mediterranean fruit fly, *Ceratitis capitata* [37–40], Oriental fruit fly, *Bactrocera dorsalis* [41, 42] and Queensland fruit fly (Qfly), *Bactrocera tryoni* [31, 43–46]. Comparative analyses have shown substantial qualitative and quantitative divergence in rectal gland volatiles among *Bactrocera* species [47, 48].

Qflies are a major horticultural pest in northern and eastern Australia and parts of Melanesia [14, 17, 49–51], and is one of the most studied tephritids with respect to pheromone gland chemistry. Sexually mature Qfly males release a blend of volatiles from their rectal glands and disperse them via rapid wing vibrations (‘calling’) to attract females [31, 52–56]. Over 100 volatile compounds have been found in the rectal glands of Qfly males, and over 30 of these have been identified [47, 57]. The most abundant compounds are aliphatic amides [43, 58, 59]. However it is not yet known which of the compounds are responsible for the short range attraction of females observed in experiments with crushed male rectal glands.

Qfly females also secrete diverse volatiles from their rectal glands, including lesser amounts of some of the amides found in males, with their profiles instead dominated by fatty acid esters [47, 57, 60, 61]. Overall a majority of the volatiles secreted from Qfly rectal glands show at least quantitative and in many cases presence/absence differences between the sexes [47]. Less is known about the functions of the volatile compounds emitted by female Qflies [62, 63], although work on other tephritids has implicated their female rectal gland volatiles in some mating and other reproductive behaviours [41, 64–66].

Qualitative and quantitative geographic variation in male pheromone composition has been described in two tephritid species, specifically between three different *C*. *capitata* populations in Greece and Argentina [67, 68] and seven different *Anastrepha fraterculus* populations in Brazil and Argentina [69]. However, the genetic basis of this variation, and any consequences for its behaviour, are not yet known. Nor is anything yet known about geographic variation in Qfly pheromone composition, although the species does show heritable geographic variation in climate stress tolerance [70] and molecular population genetic studies show clear genetic divergence between geographic populations, including geographically proscribed introgression from the closely related taxon *Bactrocera aquilonis* [50, 71].

Non-genetic factors could also generate geographic variation in rectal gland volatiles in fruit flies such as Qfly. For example, geographic variation in host plant utilisation could play a role, because host plant chemicals can be translocated to rectal glands in some tephritids, and, in certain of those cases, transformed to other bioactive volatiles [28, 31, 72–74]. Many of the fatty acid esters found in rectal glands of Qfly females are also found in host fruits [75–78] and some are attractive to Qflies of both sexes [79]. One well studied example of such a process involves cuelure (4-(4-acetoxyphenyl)-2-butanone), an analogue of the more common naturally occurring plant phenylpropanoid raspberry ketone [80]. Cuelure is a strong attractant for Qfly males (as is raspberry ketone) and is a widely used lure for monitoring this species [19, 22, 81, 82]. When ingested, cuelure is transported through the haemolymph to the rectal gland, where it is stored in its hydrolysed form as raspberry ketone and released together with the endogenously produced volatiles [73].

Testing for a genetic basis to any geographical variation in rectal gland volatiles therefore requires a ‘common garden’ approach which controls for dietary and environmental effects.

Here we report our application of gas chromatography-flame ionization detection (GC-FID) techniques to analyse rectal gland extracts of male and female Qflies from a long domesticated laboratory colony and 23 isofemale lines that were recently established using flies sourced from across this species’ geographic range. The analyses were conducted in a common garden laboratory environment with carefully defined diet to control for dietary effects. We found differences between lines across generations in the abundance of 26 compounds in males but none in females. Most of the 26 compounds were more abundant in the long domesticated stock than the isofemale lines, which is consistent with the genetic responses to selection on various other traits which have been reported to occur during domestication of this and other insect species [83–85]. However, several of the compounds also showed differences among the isofemale lines, and these included differences both within and between source populations. Some of the variation was correlated across lines, suggesting coordinated genetic control, but much of it was not, implicating variation in several different genes. Four of the 26 varying compounds were identified against authentic standards and another four were tentatively identified from the mass spectra of peaks with equivalent Kovats Indices (KIs) in parallel solid phase microextraction-coupled gas chromatography-mass spectrometry (SPME GC-MS).

## Results

### GC-FID detection of significantly varying peaks

Twenty-three isofemale lines were established from seven collections of Qflies from widely separated locations in northern and eastern Australia in 2017 and 2018 (Table 1). Sexually mature flies from single sex (i.e., virgin) and mixed sex cohorts were assayed for the nine lines collected latest in this period, referred to hereafter as the major set, whereas only males from mixed sex cohorts were assayed for the 14 earlier collected lines, hereafter referred to as the minor set. The two sets of lines were assayed in different batches but the long-domesticated S06 line, which was originally collected from Sydney in 2006 [85], was also included in both sets (albeit using different S06 generations in the two sets). The flies in the mixed sex cohorts (hereafter denoted mixed males and mixed females, respectively) were obtained from cages containing both sexes, 90–95% of females from which had been mated [47].

**Table 1:** *B*. *tryoni* lines used in the GC-FID analyses. Column heading “Screened generations” refers to the generations in the laboratory post-collection when the GC-FID analyses were carried out. Precise numbers for these generations could not be provided for S06, because records for its first few years in the laboratory were not available.

Strain type	Source population	Lines	Location		Screened generations	Collection date
			Latitude	Longitude		
Isofemale lines (major set)	Alice Springs	AS09, AS19, AS36	-23.69	133.89	6, 7, 8, 10	Nov 2017
	Cape Tribulation	CT07, CT38, CT60	-16.09	145.46	4, 5, 6, 8	Aug 2018
	Sydney	SY13, SY18, SY53	-33.90	151.14	6, 7, 8, 10	Sep 2017
Isofemale lines (minor set)	Brisbane	BR05, BR34, BR39, BR50	-27.41	152.90	6, 8, 10, 12	Mar 2017
	Mareeba	MB09, MB46, MB50, MB64	-17.01	145.43	6, 8, 10, 12	Feb 2017
	Narrabri	NB02, NB11, NB28	-30.33	149.78	6, 8, 10, 12	Mar 2017
	Utchee Creek	UT01, UT03, UT43	-17.60	145.99	6, 8, 10, 12	Mar 2017
Mass-bred laboratory strain	Sydney	S06	-33.90	151.14	~ 116–122	2006

Our GC-FID methods were the same as those of Castro-Vargas et al. [47], whose major focus was on the S06 strain. Some of their data are also used herein (Table 2). They reliably distinguished over 180 rectal gland peaks across the two sexes and identified 45 of them in parallel solid phase microextraction gas chromatography-mass spectrometry (SPME GC-MS) analyses. The relatively large number of identified peaks enabled them to identify three retention time (Rt) intervals, 4.60–10.39, 10.40–13.59 and 13.60–21 min (hereafter short, mid and long Rt peaks), which contained disproportionately high numbers of the identified short chain alcohols, esters and ketones, aliphatic amides, and fatty acid esters, respectively. These intervals corresponded to Kovats Indices (KIs) of 840–1095, 1096–1240 and 1241–2175 respectively. Castro-Vargas et al. [47] further classified their peaks as major, intermediate and minor in respect of abundance if they contributed ≥ 1%, 0.1–0.99% or < 0.1% of total peak area in the sex/mating history category where they were most abundant, and also rated them as sex specific (only present in one sex), sex selective (>log_10_ difference in average peak area between the sexes) or no strong sex bias (<log_10_ difference in average peak area between the sexes) within each mating history category.

**Table 2:** Peaks detected in the CG-FID analysis of virgin and mixed flies from the major set of lines, categorised as per Table 2 of Castro-Vargas et al. [47] by sex specificity and selectivity. Peaks shown as bolded, underlined or plain text are classified as major, intermediate or minor in abundance, respectively, in the category in which they were most abundant. “~” refers to peaks with no sex specificity or selectivity. “nd” refers to peaks not detected in the corresponding category. Seventeen other peaks only detected in minor set mixed males were not classified for sex specificity because minor set females were not assayed (see S1 Dataset).

Type	Single sex	Mixed sex	Peaks		
Female biased (34)	Female specific (21)	Female specific (12)	Short Rt	Mid Rt	Long Rt
			8.82	11.27	15.51, **18.40**, **18.46 (Ethyl tetradecanoate)**, 18.73, 18.78, 18.83, 19.59, 20.05, 20.11, 20.25
		Female selective (3)			19.14, **19.51 (Ethyl (*E*)-9-octadecenoate)**, 20.55
		~ (2)			18.61, 19.76
		n.d. (4)	9.63		18.85, 19.70, 19.99
	Female selective (10)	Female selective (7)		**10.85**	**17.55**, **17.69**, **18.88**, **18.98 (Ethyl (*Z*)-9-hexadecenoate**), **19.01**, **19.39**
		~ (2)			**18.42 (Ethyl (*Z*)-9-tetradecenoate)**, **18.93**
		n.d. (1)			20.52
	~ (3)	Female specific (2)			18.37, 20.31
		Female selective (1)			18.21 (Methyl tetradecanoate)
Male biased (110)	Male specific (63)	Male specific (49)	4.61 (*n*-propyl 2-methylpropanoate), 5.20, 5.41, 5.99, 6.30, 6.64, 6.94, 8.53, 9.43, 9.58	11.46, 11.93, **13.59 (*N*-(3-Methylbutyl)-2-methylpropanamide)**	13.79, 13.95, 15.22, 15.38, 15.67, 15.98, 15.99, 16.18, 16.52, 16.59, 16.66, 16.85, 16.89, 16.94, 17.01, 17.07, 17.17, 17.26, 17.41, 17.64, 17.75, 17.84, 18.01, 18.48, 18.68, 18.80, **18.89**, 19.08, 19.12, 19.62, 19.72, 19.83, 20.33, 20.59, 20.64, 20.98
		n.d. (10)	10.05	**13.33**	16.73, 17.72, 18.76, 19.04, 19.65, 20.19, 20.35, 20.81
		~ (4)			15.59, 18.72, 20.01, 20.13
	Male selective (4)	Male specific (2)		10.41 (*N*-(2-Methylbutyl)acetamide), 11.04	
		Male selective (2)		**10.59 (*N*-(3-Methylbutyl)acetamide)**, **12.96 (*N*-(3-Methylbutyl)propanamide)**	
	~ (14)	Male specific (12)	4.82 (4-Heptanone), 4.96, 6.21	12.27	15.87, 17.15, 17.32, 17.52, 18.12, 18.53, **19.48**, 20.07
		Male selective (2)		**12.63 (*N*-(2-Methylbutyl)propanamide)**	**19.43**
	n.d. (29)	Male specific (29)	4.07, 7.79, 8.73, 9.79, 9.98	11.7, 11.79 (Diethyl succinate),11.97, 12.45, 13.34	13.88, 14.54, 15.07, 15.45, 16.14, 16.70, 16.78, 18.02, 19.15, 19.20, 19.31, 19.78, 19.92, 19.94, 19.97, 20.22, 20.23, 20.40, 20.42
Changed specificity (4)	Male specific (2)	Female specific (2)			18.39, 20.62
	Female specific (2)	Male specific (2)			15.63, 20.15
No sex bias (36)	~ (31)	~ (31)	4.23, 6.06, 7.66 (2-Ethyl-1-hexanol), 8.38	**13.47 (*N*-(2-Methylbutyl)-2-methylpropanamide)**	14.37, 14.86, 15.77, **16.11**, 16.32, 16.45, 17.21, 17.37 (Methyl dodecanoate), 17.45, 17.63 (Ethyl dodecanoate), 17.92, 17.96, 18.10, 18.17, 18.27, 18.32, 18.64, 19.25, 19.29, 19.35, 19.56, 19.66, 19.80, 19.86, 20.50, 20.84
	n.d. (5)	~ (5)			16.50, 20.37, 20.68, 20.88, 20.90

We recovered 184 of the peaks of Castro-Vargas et al. [47] in our data sets and their Rt ranges and abundance and sex bias classifications are summarised in Table 2. Seventeen additional peaks not reported by Castro-Vargas et al. [47] were also detected in our minor set of lines; these were all rated as minor in abundance in the mixed males we analysed from these lines but in the absence of data for females from these lines they could not be classified for sex bias. The four abundance data sets (i.e., for virgin and mixed flies of each sex) for the major set of lines are in S1 Dataset of Castro-Vargas et al. [47], and the one abundance data set (i.e. for mixed males only) for the minor set of lines is in S1 Dataset of this article. S1 Fig shows illustrative GC-FID chromatograms.

**Fig 1:**
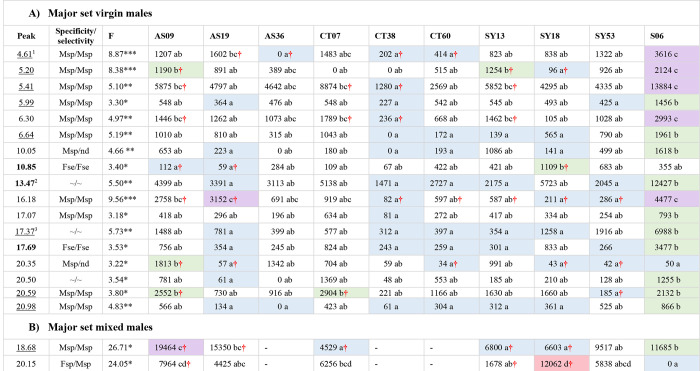
Back-transformed emmeans (estimated marginal means) for peak areas for peaks showing significant differences among isofemale lines in the major set of virgin (A) and mixed (B) males. As per Table 2, peaks are bolded, underlined or neither if they are classified as major, intermediate or minor in abundance. Known peak identities are indicated with superscripts as follows: ^1^*n*-propyl 2-methylpropanoate; ^2^ *N*-(2-methylbutyl) 2-methylpropanamide; ^3^ methyl dodecanoate. Sex bias (also taken from Table 2) is indicated as follows: Msp = male specific, Mse = male selective, Fse = female specific, Fse = female selective, nd = not detected and ~ = not sex biased, with the bias in virgin and mixed sex cohorts indicated before and after the slash, respectively. Bold lines between rows separate peaks in the different Rt ranges. Confidence limits are given in S2 Table (see Materials and Methods). FDA-corrected significance values for F statistics are indicated with asterisks, with * p < 0.05, ** p < 0.01 and *** p < 0.001. Letter codes are used to show the results of *post hoc* pairwise contrasts between lines, where lines with the same letters are not significantly different from one another. Lines with a single letter only sit in a single group of lines and non-overlapping lines/groups of lines are coloured, with blue, green, purple and pink representing groups with progressively higher values. A red **†** indicates a value in an isofemale line which differs significantly from the value(s) in at least one other isofemale line(s). Some apparent inconsistencies between the groupings reflect variation in the between-replicate variances for the particular means, as evidenced in S2 Table. A dash indicates the peak was not analysed in that line. Replicate numbers are indicated in S1 Dataset of Castro-Vargas et al. [47].

**Fig 2:**
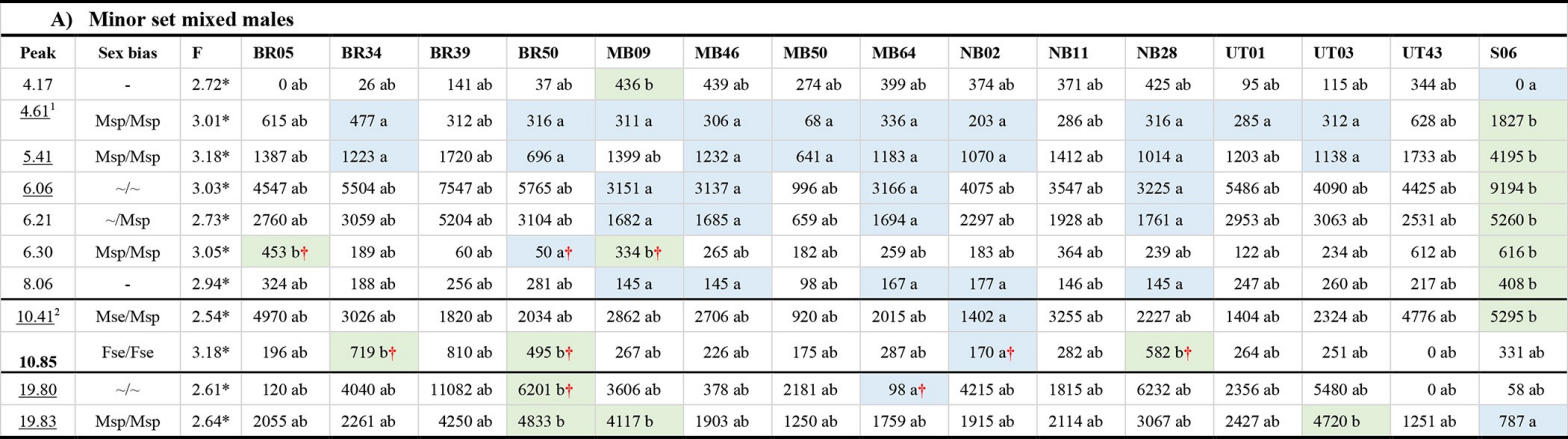
Back-transformed emmeans for peak areas for peaks showing significant differences among minor set lines for mixed males. Formatting is as per Fig 1 and confidence limits are given in S2 Table. Known peak identities are indicated with superscripts as follows: ^1^*n*-propyl 2-methylpropanoate; ^2^*N*-(2-methylbutyl) acetamide. Note that sex bias data are not shown for peaks 4.17 and 8.06 because they did not meet the inclusion criterion (detected in > 50% of samples in at least one sex/mating history category) for analysis in the major set of lines and therefore lacked data for females. As per Fig 1, some apparent inconsistencies between the groupings reflect variation in the between-replicate variances for the particular means. Replicate numbers are indicated in S1 Data set attached.

Linear modelling (see Materials and Methods) of abundance data, as represented by peak areas, from each of the five data sets found no significant variation among the constituent lines in any of the peaks in either female data set (i.e., the major virgin and mixed female sets; 85 and 75 peaks tested, respectively) but found a total of 30 cases of significant variation among lines across the three male data sets (17 cases in major set virgin males, two in major set mixed males and 11 in minor set mixed males; 127, 153 and 120 peaks tested, respectively). S1 Table shows the linear modelling results for all peaks tested and Figs 1 and 2 summarise the abundance data for each line for the 30 cases of significant variation, with the corresponding confidence limits given in S2 Table. Twenty six peaks were involved in the 30 cases of significant differences, with four peaks (4.61, 5.41, 6.30 and 10.85) showing significant differences in both the major set virgin and minor set mixed males. Greater statistical power due to higher sampling intensity likely contributed to the higher numbers of significant differences seen in the major set virgin males and minor set mixed males but it is not clear whether the absence of any significant differences in the two female data sets was solely due to their lower sample sizes, since two differences were found in the similarly sized sample of major set mixed males (see Materials and Methods). The 26 significantly varying peaks represented 14% of all peaks tested across the three male data sets.

### GC-FID characterisation of significantly varying peaks

Four of the 26 peaks involved in significant lines differences had been identified in Castro-Vargas et al. [47]: 4.61 (*n*-propyl 2-methylpropanoate), 10.41 (*N*-(2-methylbutyl)acetamide), 13.47 (*N*-(2-methylbutyl)-2-methylpropanamide) and 17.37 (methyl dodecanoate). Sex specificity was likewise already known for 24 of the 26 (all but two that were significant in minor set mixed males but not detected in the major set) and over half of these (14) were male-specific. Three of the 26 were ranked major in abundance, 13 as intermediate and ten as minor, which was similar to the ratio of the three abundance categories among all peaks tested across the five data sets (21: 75: 105, respectively; χ^2^ = 2.36, df = 2, p > 0.05).

However, the ratio of significant peaks in the short, mid and long Rt ranges, 11: 3: 12, respectively, differed from that of 31: 23: 147, respectively, seen among all the peaks tested across the five data sets (χ^2^ = 14.77, df = 2, p < 0.001). In particular there was a marked excess of significant peaks in the short Rt range, and a corresponding deficit of those in the long Rt range. Thus, 35% of the short Rt peaks tested differed significantly between lines and, given that three of the four peaks that varied significantly in two data sets had short Rt’s, they accounted for 47% of the 30 cases of significant differences among lines.

*Post hoc* pairwise comparisons using emmeans (Figs 1 and 2, S2 Table and see Material and Methods) showed that the long domesticated line, S06, had the highest abundance value for 20 of the 30 cases of significant line differences. In particular, S06 had the highest value for 13 of the 14 cases involving short Rt peaks. In some cases, the values for S06 were about two-fold, and in a few cases three- or four-fold, higher than the highest value among the isofemale lines. Interestingly, in four cases, including the one exception among the short Rt cases where S06 did not yield the highest value, it actually recorded amongst the lowest values. In two of these, including the exceptional short Rt case, the peak was undetectable in S06.

In addition to the differences involving S06, there were also significant differences among the isofemale lines in 13 of the 30 cases, five involving the short Rt range, two the mid Rt range and six the long Rt range (Figs 1 and 2 and S2 Table). A total of eleven peaks contributed to these cases, with two peaks (6.30 and 10.85) differing in both the major set virgin and minor set mixed male data sets. Six of the 13 cases involved four- to ten-fold differences between the highest and lowest lines for these peaks, the rest all being greater than 20 fold. Two peaks otherwise classified as intermediate in abundance were undetectable in certain line(s). While there was some trend for particular lines to have either relatively high (e.g., AS09 and CT07) or relatively low (e.g., CT38) abundances across multiple peaks, most of the lines showed a mix of relatively high and low abundance values. The correlations across peaks are assessed in more detail below.

All nine of the isofemale lines in the major set showed significant differences between them in pairwise contrasts in at least one peak in the virgin males and all but one of those tested also did among the mixed males (red †s in Fig 1 and see also S1 Table). One of these major set lines, AS09, differed significantly from some of the others in nine different peaks. In contrast, only half of the 14 minor set isofemale lines showed significant differences in at least one peak and the highest number of peaks involved in a significant difference in a single minor set line was two, for BR50 (red †s in Fig 2 and see also S2 Table). Only one source location, Utchee Creek, in the minor set of lines, was not involved in any significant differences between any of the isofemale lines. Furthermore there were significant differences between isofemale lines from the same source location for all locations except Utchee Creek and Mareeba, also in the minor set of lines.

In order to assess higher level differences in the composition of the rectal gland volatiles among lines, we then used permutational multivariate analyses of variance (PERMANOVAs) to investigate the extent of overall differences between the lines across all the peaks in each of the three Rt ranges. Thus these analyses used the data for all 201 peaks, not just the 26 which had individually shown significant differences. The PERMANOVAs revealed significant differences among both virgin and mixed males in the major set of lines, as well as in mixed males from the minor set, but not in either virgin or mixed females from the major set (Table 3). Differences in the major set virgin males involved both the short and mid Rt ranges; those in the major set mixed males involved the mid and long Rt ranges; and those in the minor set mixed males involved all three Rt ranges. Several of these differences were largely due to S06 being different from the other lines because they became non-significant when the S06 data were excluded from the analyses (Table 3). However, there were also significant differences between isofemale lines from different source localities for major set mixed males for peaks in the long Rt range, and for minor set mixed males for peaks in the short Rt range (Table 3).

**Table 3:** Summary of PERMANOVAs assessing overall cross-peak differences in each of the three Rt ranges in rectal gland volatiles in the five data sets. Analysis I assessed the differences across all 24 lines. If that term was significant, Analysis II, which excluded S06, then assessed the differences due to locality of origin and lines within localities. F ratios are shown with degrees of freedom in brackets. * p < 0.05; ** p < 0.01; *** p < 0.001.

	Major set				Minor set
	Virgin males	Mixed males	Virgin females	Mixed females	Mixed males
Analysis I: Differences across all 24 lines for each Rt range					
Short Rts; Lines	2.40 (9,37)***	2.60 (6,6)	1.21 (9,17)	4.5 (6,4)	1.60 (14,88)**
Mid Rts; Lines	2.07 (9,37)*	4.60 (6,6)*	0.90 (9,17)	2.20 (6,4)	1.60 (14,88)*
Long Rts; Lines	1.30 (9,37)	5.00 (6,6)*	0.95 (9,17)	0.60 (6,4)	1.30 (14,88)*
Analysis II: Differences across the 23 isofemale lines for each Rt range					
Short Rts; Localities	1.80 (2,32)	-	-	-	2.93 (3,83)***
Short Rts; Lines within Localities	1.44 (6,32)	-	-	-	0.81 (10,83)
Mid Rts; Localities	1.02 (2,32)	3.60 (2,4)	-	-	0.44 (3,83)
Mid Rts; Lines within Localities	1.41 (6,32)	0.09 (3,4)	-	-	1.52 (10,83)
Long Rts; Localities	-	8.90 (2,4)*	-	-	1.01 (3,83)
Long Rts; Lines within Localities	-	0.17 (3,4)	-	-	1.21 (10,83)

**Fig 3:**
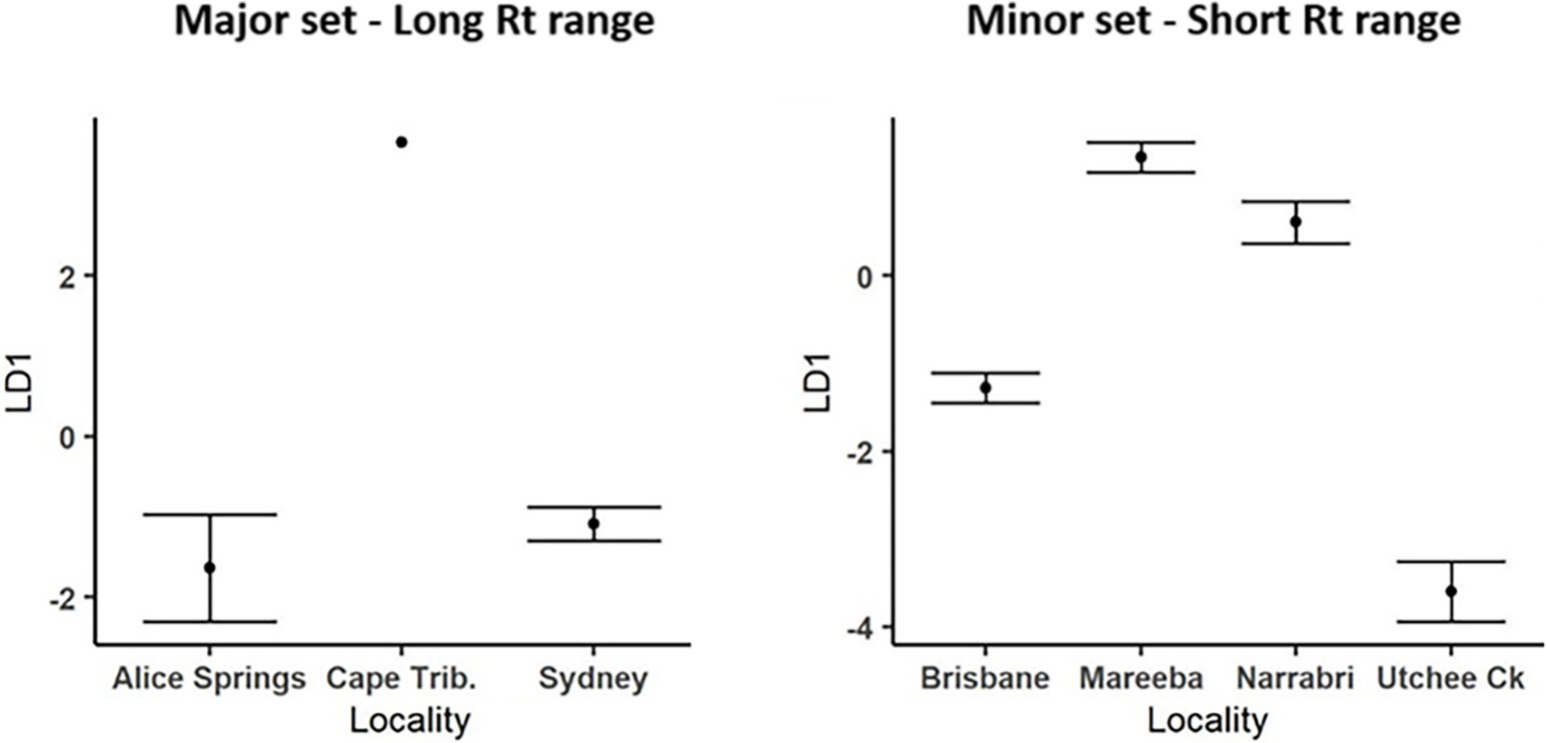
LDAs of significant cross-peak differences between localities in the PERMANOVAs in Table 3. Separate plots are shown for peaks from major set mixed males eluting in the long Rt range and those from minor set mixed males eluting in the short Rt range. Standard error bars are also shown. LD1 in each plot explained > 80% of the between-locality variance.

Linear Discriminant Analysis (LDA; Fig 3) showed the difference in the major set mixed males in the long Rt range was largely due to Cape Tribulation differing from Sydney and Alice Springs, but this result was difficult to interpret because only a single Cape Tribulation line had sufficient data to be included in this analysis. On the other hand, the LDA for the short Rt peaks in the minor set mixed males clearly separated the four populations into three groups, with Mareeba plus Narrabri at one extreme, Utchee Creek at the other, and Brisbane between them.

**Fig 4:**
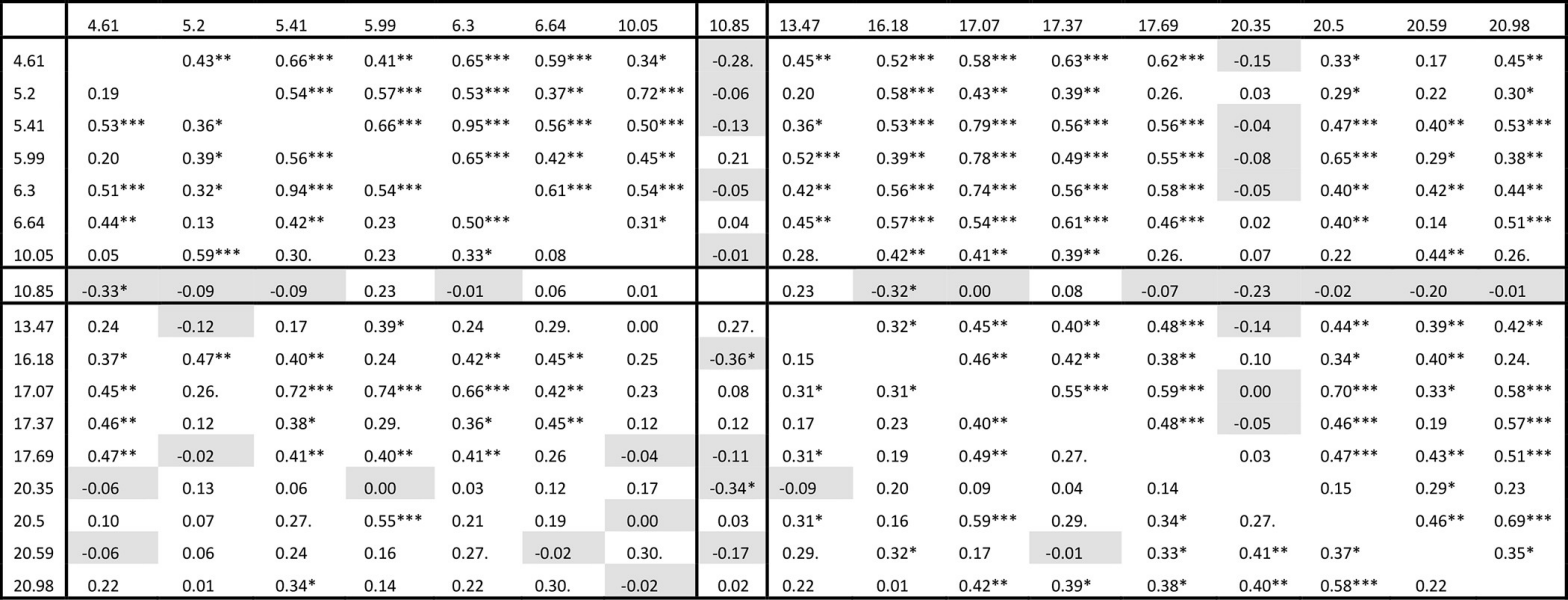
Pairwise Spearman correlation coefficients between the mean peak areas of peaks differing significantly among S06 and the major set of isofemale lines in virgin males. Raw data are taken from Fig 1. Top right and bottom left show the correlations with and without the S06 data, respectively. Bold lines between rows and columns separate peaks in the different Rt ranges. * p < 0.05, ** p < 0.01, *** p < 0.001. Negative correlations are highlighted in grey.

We also assessed the extent to which different peaks showing significant line variation showed correlated patterns of variation. To do this we focussed on a single data set, major set virgin males, because it had the most peaks showing significant differences and we wished to avoid the possibility of producing spurious patterns that might arise if we combined data sets involving different mating histories. Overall, we found many significant correlations when S06 was included in the analysis, and some of these remained significant when S06 was excluded (Fig 4). The great majority of the correlations were positive, and many of them significantly so: 95 when S06 was included and 50 when it was not. Only four of the few negative correlations across the two analyses were significant, all four involving peak 10.05 and two also involving peak 16.18. Two peaks, 10.85 and 20.35, showed little correlation with other peaks in either analysis, suggesting independent genetic control of the variation in those peaks. The analysis excluding S06 also found several other peaks were largely uncorrelated with one another, but strong correlations remained among most of the six short Rt peaks analysed, among a few of the eight long Rt peaks, and, intriguingly, between two long Rt peaks (17.07 and 17.69) and four of the short Rt peaks. The strong correlations suggest some shared genetic basis for the differences in the peaks involved, both among the isofemale lines and between them and S06. Overall, however, low r^2^ values show that most of the variation in different peaks was independent of those in other peaks.

### GC-MS characterisation of significantly varying peaks

SPME GC-MS analysis of samples of virgin and mixed S06 males and females was then carried out in order to further characterise the 22 significantly varying peaks that had not already been identified against authentic standards in our GC-FID analysis. The gas-liquid extraction methods mediated by the SPME fibres yield cleaner mass spectra, albeit not always the same peaks, as compared to the liquid-liquid hexane extractions used in our original GC-MS analysis [47].

Our strategy for this work was first to establish the overall relationship between the Rt profile of the peaks detected by GC-FID and the profile of Kovats Indices (KIs) obtained for peaks detected by the GC-MS analysis, which we did by comparing the results of peaks identified against authentic standards with both technologies. This relationship could then be used to impute KI values for all the other GC-FID peaks. Further, by comparing the imputed KIs of the other GC-FID peaks with the observed KIs of the SPME GC-MS peaks, and also considering their relative abundances in virgin and mixed flies of each sex, we were able to pair more GC-FID peaks with GC-MS spectra than had originally been possible just using the original GC-MS data of Castro-Vargas et al. [47] and the relatively small number of shared authentic standards. These additional pairings enabled us to assign mass spectra to the respective GC-FID peaks and, in several cases, to assign identities to them. In some cases confirmed identification were possible, because of the greater number of authentic standards used in the GC-MS work. In other cases tentative identifications could be made in the absence of authentic standards because the spectra were of sufficient quality to recognise matches in the National Institute of Standards and Technology (NIST) database.

The SPME GC-MS analyses revealed a total of 97 peaks across a KI range from 674 to 2188 (S2 Dataset). This included all of the 37 peaks we had previously identified with authentic standards (S3 Table). Fifteen peaks (hereafter termed calibrator peaks) of these 37 had also been identified using the corresponding standards in the GC-FID analysis (Table 2). Calibrations of the Rt data from the GC-FID analysis against the KI data from the GC-MS analysis for these 15 (S2 Fig) then allowed us to impute KIs for all 201 of the GC-FID peaks analysed (S1 Table).

We then compared the KI profiles of the GC-FID and GC-MS peaks in the KI interval from 839 to 1783 which was common to both data sets (S4 Table). This region contained 105 GC-FID and 50 GC-MS peaks in male samples, including eight of the 15 calibrator peaks. The 105 GC-FID peaks included 14 of the previously unidentified peaks that had varied significantly between lines. Thirty five of the 105 male GC-FID peaks, including six of the latter 14, were provisionally assessed as being common to the two data sets on the basis of their KI values.

The identities of nine of these 35 (the eight calibrator peaks plus one other peak) were already known because they had been validated with standards in the GC-FID analyses in Castro-Vargas et al. [47]. Another seven of the 35 were discounted as potentially false matches because of differences between their relative abundances across the four sex/mating history categories as calculated from their peak areas in the GC-FID and SPME GC-MS analyses (S4 Table; note that quantitative data had not been collected in the original GC-MS experiment). The latter was a harsh filter because the differences in extraction techniques (the GC-FID, like the original GC-MS data, were based on liquid-liquid hexane extractions) would be expected to affect recovery efficiency anyway, and indeed such differences were seen in some of the eight shared calibrator compounds. Nevertheless, it was considered necessary in order to minimise the risk of false positive matches. It left us with 19 previously anonymous GC-FID peaks in males for which the GC-MS matching then enabled us to confidently assign mass spectral data (S3 Fig). This included all six of the peaks that had shown significant variation between lines above.

Five of the 19 previously anonymous GC-MS-matched GC-FID peaks could also then be identified because the corresponding standards had been included in the original GC-MS analysis (Table 4). They included a short chain alcohol, an aldehyde and an ester in the Short Rt range and an amide and a ketone in the Mid Rt range. Amongst these peaks were three of the six in the shared set that had shown significant variation between lines, namely *n*-octen-1-ol, an *x*-octenal isomer and ethyl 2-methyl pentanoate, none of which have previously been implicated in insect pheromone functions.

**Table 4:** Details of 19 pairs of GC-FID and GC-MS peaks matched for KI and sex-specific abundances in S06 males. Six of these peaks which showed significant differences among lines in Figs 1 or 2 are indicated with asterisks. The Rts, GC-FID KIs, GC-MS KIs and confirmed or tentative identifications are given. As per Table 2, Rts for peaks classified as intermediate in abundance are underlined while those rated minor in abundance are not. None of the 19 peaks were rated as major in abundance. See S3 Fig for the mass spectra for these 19 peaks and S1 Table for the imputed KIs of all the other GC-FID peaks. As S4 Table shows, a twentieth GC-FID peak, Rt 18.42 and imputed KI 1783, was matched to a GC-MS peak but it had already been identified from a standard in the GC-FID runs and was unavailable for use as a GC-MS standard.

Rts	KI(imp)	KI(obs)	Validated identifications	Tentative identifications
5.20⁕	885	883		2-Methyl-3-hexanol
6.21⁕	933	933	Ethyl 2-methylpentanoate	
6.30⁕	937	939	*x*-Octenal isomer 2	
6.64	953	955		*n*-Butylcyclopentane
7.08	974	972		Phenol
8.06⁕	1021	1019	*n*-Octen-1-ol	
9.58	1093	1094	N-(2-Methylpropyl) propanamide	
10.05⁕	1115	1115		
11.16	1161	1164		
11.46	1171	1171	2-Bornanone	
11.5	1172	1173		
11.7	1179	1179		Borneol isomer 1
11.93	1185	1187		Borneol isomer 2
11.97	1186	1188		
13.88	1252	1253		
13.95	1254	1256		
14.37	1267	1267		
17.07⁕	1463	1464		
17.17	1482	1484		

Detailed examination of the mass spectra for the other 14 previously anonymous GC-MS-matched GC-FID peaks and comparisons to the NIST library then yielded a further five identifications, which, in the absence of authentic standards, were nevertheless designated as tentative (Table 4). These comprised two more alcohols and an alkane in the short Rt range and two alcohols in the mid KI range. One of the short Rt alcohols, 2-methyl-3-hexanol, was another one of the six in the shared set that had shown significant variation between lines. This compound has not been implicated in insect pheromone functions in previous literature.

One of the other four newly identified compounds, the short Rt alcohol, phenol, may just be a trace metabolite of a more complex phenolic compound(s) because phenol itself is toxic. However, various phenolics, and phenol itself, have been implicated in pheromone functions in other insects and ticks [86, 87]. The two newly identified mid Rt alcohols are also notable because both are isomers of borneol and closely related to 2-bornanone, a.k.a. camphor, which we had previously identified in S06 rectal glands [47]. Camphor and borneol have both been implicated in sex pheromone functions in another insect [88].

None of the GC-MS peaks had a KI (~1462) or mass spectrum resembling raspberry ketone. This concurs with previous literature which has only recorded the compound in the rectal glands of Qfly laboratory cultures when they are provided it in their diet [31, 73].

## Discussion

This appears to be the first study to use isofemale lines to identify inherited intraspecific variation in any tephritid phenotype. The technique has been widely used to detect inherited trait variation in wild populations of *Drosophila* (e.g., [89, 90]) and recently also mosquitoes [83]. It effectively captures and preserves polymorphism in recently collected field populations that might otherwise be lost during domestication. It does this by trapping genetic variants in different lines each established from a different single mated female. In our case, we have then used repeat assays of these lines over multiple generations to confirm the genetic basis of differences in a phenotype which has previously been refractory to genetic analysis in tephritids. While our results clearly show genetic variation between lines reared and tested in a common garden environment, they do not of course exclude the possibility that diet can also contribute to variation in the field. Future work will be able to use crosses among isofemale lines to further dissect the genetics involved, including by modern Quantitative Trait Locus (QTL) analyses. We suggest the isofemale line approach can become an important tool for genetic analyses of diverse traits in tephritids, providing valuable information both for understanding their population biology as well as to guide breeding programs for, for example, SIT mass release strains (see below).

In our case, the limited replication possible in the early post-collection generations of some of the isofemale lines would have limited our power to detect significant line differences in abundances. Nevertheless, we have still found quantitative genetic variation in 26 individual male peaks, representing ~14% of all peaks scored in males, among our isofemale lines and the long domesticated S06 line. Some of the differences were manifest in virgin males, some in mixed males and four in both (albeit, in the latter case, not in the same lines). Notably, just over half of the variable male peaks were male-specific. By contrast, no differences were found among females from either single or mixed sex samples. This accords with the findings of Castro-Vargas et al. [47] who found presence/absence differences between the sexes in ~ 46% of the 184 peaks they analysed, and quantitative differences between the sexes in another ~ 34%, with many more of both categories being male-specific or -selective than female-specific or -selective (see also Table 2). The results of both studies thus suggest profoundly different biological functions for the rectal gland volatiles of the two sexes.

The 26 peaks showing significant differences in males covered a wide range of Rt values but a disproportionately high number, eleven, were in the short Rt range (Figs 1 and 2). Given only 31 short Rt peaks in total were assayed, over a third of them varied between the lines. Short Rt peaks would be expected to be more volatile than other peaks and therefore perhaps candidates for longer range sexual attraction or other pheromonal functions. Nine of the ten variable short Rt peaks whose sex specificity/selectivity had been determined were male-specific, which would support the idea that they have a role in male sex pheromone functions. None of the eleven were rated major but five were intermediate in abundance. We were able to identify five of the eleven, namely the esters *n*-propyl 2-methylpropanoate and ethyl 2-methyl pentanoate, the alcohols 2-methyl 3-hexanol and *n*-octen-1-ol, and an octenal. The latter two are known natural products and all five have been used as fruity flavour and/or fragrance additives [91–95].

Only three compounds in the mid Rt range varied between lines, although two of them were identified, *N*-(2-methylbutyl) acetamide and *N*-(2-methylbutyl) 2-methylpropanamide. Neither of the amides nor the unidentified Rt 10.85 (imputed KI 1151) were male-specific. The first amide was intermediate in abundance but the other two compounds were both rated major in abundance. The two amides were among the six aliphatic amides which have been consistently detected in previous studies of Qfly rectal glands and, in large measure because of their abundance, the amides as a group have been proposed to be male sex pheromones [43–46]. Given that we found only one of the group, *N*-(3-methylbutyl) 2-methylpropanamide, to be male-specific, this appears unlikely to us. On the other hand, in light of the major abundances of one of the amides and Rt 10.85, their variation, which in the case of the latter was evident in both sets of lines, may impact on some other function(s).

Although twelve long Rt compounds were found to vary significantly among lines, they represented fewer than 10% of all long Rt compounds scored. One of the twelve, Rt 17.69, imputed KI 1606, was major, six others intermediate, and five minor in abundance. Only one of the twelve was identified, methyl dodecanoate, which showed no sex bias [47]. Methyl dodecanoate has been found in the labial glands of several species of bumblebees, where it acts as a male sex pheromone [96–99], and it is also present in cuticular profiles of southeast Asian stingless bees [100]. Notably seven of the other variable long Rt compounds were male-specific, whereas none of the eight fatty acid esters whose identity was established by Castro-Vargas et al. [47] in the long Rt ranges were male-specific. Thus at least some of the seven unidentified variable long Rt compounds may not have been fatty acid esters either. Long chain alcohols and alkanes would also be expected to have retention times in this range and have been implicated in various pheromone functions in other insects, either in themselves or as intermediates in the biosynthesis of the active compounds [101].

Totalled across the three Rt ranges, the proportions of the 26 varying peaks that were classified as major, intermediate and minor in abundance (3:13:10, respectively) were not significantly different from the proportions among all the peaks scored. While it is tempting to argue that their relative abundances make the variability of the major and intermediate compounds more relevant biologically, Jang et al. [38] found no relationship between the abundance of several rectal gland volatiles and their biological activity as measured by electroantennogram responses in *C*. *capitata*.

Both our individual volatile analyses and multivariate analyses found significant differences in male volatiles between the isofemale lines as a group and the domesticated S06 line. Most of the significantly varying peaks in the individual volatile analyses were either substantially more abundant in S06 than any of the isofemale lines or, in a smaller number of cases, amongst the very lowest abundances seen amongst the isofemale lines (and in two cases no longer detectable). Peaks in all three Rt ranges were implicated in the abundance differences between S06 and the isofemale lines. Notable also is the fact that three of the isofemale lines were from Sydney, the same locality as S06. Insomuch as two of the peaks that were higher in S06 included the two significantly varying amides, our data also bear out an earlier finding [58] that the amounts of some of the aliphatic amides increased during domestication. Taken together, all these results suggest a strong but complex pattern of directional selection has operated on many of the rectal gland volatiles during the domestication of S06. It is perhaps to be expected that the high densities in which most laboratory colonies are maintained would select for different male pheromone compositions [68]. The effect could potentially explain some of the relatively low mating success of mass released sterile males in the field that has been observed in SIT programs for various tephritids [102].

Our analyses have also found differences between isofemale lines both within and across their source localities. The analysis of individual peaks showed peaks in all three Rt ranges and all three abundance categories were involved and the variation within localities in a few cases involved order of magnitude differences. Interestingly, most of the lines in the major set were involved but only about half the minor set lines were involved, and differences between localities were also more pronounced in the major than minor set lines. This may be because the three localities sampled in the major set isofemale lines included the most westerly (Alice Springs), northerly (Cape Tribulation) and southerly (Sydney) populations that we scored. Notably, however, the multivariate analyses of all peaks in the male data sets, whether or not they individually showed significant differences, was also able to differentiate lines from different localities in the minor set that had not been distinguished by the individually varying peaks. In general terms, our findings are consistent with earlier work showing significant geographic variation in desiccation resistance [70] and some genetic and genomic markers [50, 71, 103, 104] in Qflies. Our data thus also support the conclusion from those earlier studies that there is significant ecotypic variation among Qfly populations. Some of the variation we have found may reflect the significant but variable levels of introgression of genes from the sister taxon *B*. *aquilonis*, which is most prevalent in Qflies from the north-western parts of its range [71].

Significant positive correlations were found between several of the 17 peaks that showed significant variation across lines in virgin males from the major set (Fig 4). This was particularly the case when S06 data were included in the analysis but remained so in several cases when S06 data were excluded. Although r^2^ values seldom exceeded 0.5, these correlations imply some shared genetic basis for the variation in the peaks involved. The correlations were strongest between some of the short Rt peaks, between some of the long Rt peaks and, in a small number of cases, between short Rt and long Rt peaks. Interestingly, some of these patterns may be interpretable in terms of the likely biosynthetic pathways for peaks in the different Rt ranges [47, 101]. For example, the long Rt peaks, many of which are known or likely to be fatty acid esters, alcohols, acids and alkanes, are likely to have been generated from acetyl CoA precursors, so some could have shared precursors. Conversely, the available data indicates some of the amides in the mid Rt range are produced by an entirely different process, involving condensation reactions between amino and other organic acids, which might explain why peaks in this Rt range showed relatively few correlations with those in the other ranges. On the whole, however, the conclusion from the correlational analysis is that most of the variation in most peaks had independent genetic bases, implying that variation in multiple genes contributed to the line differences in the volatile contents of virgin male rectal glands.

Overall our data are consistent with the hypothesis [47, 105] that inherited variation exists in male sex pheromones that may facilitate speciation in tephritids. More direct support for this hypothesis will now require a) further GC-MS work to identify some of the 18 still anonymous significantly varying peaks, b) electrophysiological and behavioural tests to assess the functional significance of particular variations, and c) mapping and identification of the key gene(s) involved. While beyond the scope of this article, all three of these objectives are feasible in the near term future.

Expanding the number of identified varying peaks could be achieved by more extensive GC-MS, including use of more extraction techniques (e.g., different SPME fibres [57]) and authentic standards. Our panel of standards was depauperate for long chain alkanes and alcohols, fatty acids and terpenes, which have recurrently been implicated in pheromone functions in various insects [101], as well as spiroacetals, which have been implicated in various such functions in tephritids in particular [32, 33, 106, 107].

Electrophysiological assays focusing on both the antennae and palp are now well established for various bactrocerans [32, 33, 38, 66, 107]. Likewise, behavioural assays in various formats and spatial scales have been developed for measuring different aspects of the attraction response. Results for a range of tephritids with these various formats suggest that short *vs* long distance attraction, attraction *vs* retention (arrestation) and opposite-sex *vs* same- or both-sex attraction/aggregation could each involve distinct pheromones [36, 38, 40, 106, 108, 109], and hence have distinct genetic bases.

The genetic aspects of this subsequent work would be facilitated by the availability of genome assemblies for *B*. *tryoni* (GenBank accessions GCA_000695345.1 [110] and GCA_016617805.2 (RefSeq)) and the rapidly decreasing costs of full genome resequencing of multiple individuals. Also useful in this respect, our results suggest that a single cross between a domesticated and wild/caught line could simultaneously capture variation in many different volatiles and enable joint mapping of multiple different encoding genes.

A further priority will be to investigate differences in the rectal gland compositions and the key genes controlling them in the two closest relatives of Qfly, namely the Northern Territory fruit fly *B*. *aquilonis*, with which some hybridisation has occurred [71], and the lesser Queensland fruit fly *B*. *neohumeralis*, for which there is as yet no evidence of hybridisation in the field, despite extensive sympatry and the fertility of crosses with *B*. *tryoni* in the laboratory [15]. Genome sequence is also available for *B*. *neohumeralis* (https://www.ncbi.nlm.nih.gov/data-hub/genome/GCF_024586455.1/).

Finally, the behavioural and genetic analyses which we argue are priorities for further work could also be important to future efforts to improve the efficiency and effectiveness of SIT. Specifically, such analyses could enable marker-assisted breeding during the development of the mass-release strains that minimises the loss of mating competitiveness and ecological competence when the males are released into the field.

## Materials and methods

### Construction of the isofemale lines

The seven mass-bred populations from which the isofemale lines were made were established from infested fruits brought back to the laboratory between 2017 and 2018 (Table 1). Three days after eclosion, flies identified as *B*. *tryoni* following the taxonomic key of Drew [13] were used to establish laboratory colonies. Table 1 summarises the collection details for these populations. Further information on them is available in Popa-Báez et al. [70] and Ahmed et al. [111].

Isofemale lines were set up from the mass-bred stocks after one (minor set) and five (major set) generations in the laboratory. Single gravid females from each stock were placed in individual translucent polystyrene containers (425 mL, Dowlings, Canberra, Australia) and allowed to oviposit into a 35 mL plastic sauce bottle (Sistema, New Zealand) that was pierced with small holes and contained a piece of mini capsicum as an attractant to encourage localized egg laying. This oviposition device proved to be critical in boosting egg laying sufficiently to sustain the lines. After 24 h, eggs were collected from the bottle walls by rinsing them with water and placed into 250 mL plastic containers (250 mL, International Scientific Group, UK) containing gel larval diet [112]. The progeny from each female were then used to establish an isofemale line. A total of 23 lines were set up and maintained as described previously [70], except for our use of the mini capsicum as an oviposition substrate.

Extracts of flies from up to four generations were assayed by GC-FID for both the major (generations 6, 7, 8 and 10 post-collection) and minor (generations 6, 8, 10 and 12 post-collection) set isofemale lines (Table 1, S1 Dataset from Castro-Vargas et al. [47], S1 Dataset for this article). Generally only one replicate extract of glands from ten flies could be taken from each generation but occasionally up to four, all taken from different cages, were available for a particular line. On the other hand, for some lines, only one generation produced enough flies to generate a sample without risking the ongoing maintenance of the line. This was because the productivity of recently collected Qfly strains is generally low [84, 85, 113], likely exacerbated in our case by the inbreeding incurred by founding each line from a single female. The constraints were greatest for the major set isofemale lines where we were seeking four sex/mating history combinations but were reluctant to harvest females in particular until we could see larvae for the following generation. The average numbers of replicates assayed, summed across the different generations, from each isofemale line were 4.7, 1.6, 2.1, 1.1 and 7.0 for the major set virgin and mixed males, major set virgin and mixed females and minor set mixed males, respectively. The corresponding numbers for S06, being thoroughly domesticated and not isofemale, were more consistent, 6, 3, 2, 2 and 6 respectively, and, in the case of the minor set, only required two generations of sampling.

### GC-FID methods

The methods for sample preparation for the GC-FID analysis were as in Castro-Vargas et al. [47]. Briefly, flies aged 15–20 days since emergence were frozen at -20 ⁰C and their rectal glands excised under a stereoscopic microscope (Leica Microsystems, Heidelberg, Germany). Groups of ten glands were collected in 2 mL glass vials (Shimadzu Corporation, Kyoto, Japan) and immersed in 200 μL of high performance liquid chromatography grade *n*-hexane for 10 min. The hexane was then carefully transferred to 2 mL GC vials with 250 μL glass inserts (Shimadzu Corporation, Kyoto, Japan). The vials were then capped and stored at -80°C until used.

The GC-FID analyses were also carried out as per Castro-Vargas et al. [47]. Briefly, 1 μL samples were injected into an Agilent 7890A gas chromatograph (Agilent Technologies, Santa Clara, USA) fitted with a HP-5ms UI column (30 m x 0.250 mm i.d. x 0.25 μm film thickness; Agilent Technologies) using a split mode of 1/10 and helium as carrier gas at a constant flow rate of 1 mL/min. The oven temperature was initially held at 60°C for 2 min and then increased, first to 100°C at 10°C/min with a 3 min hold, then to 325°C at 5°C/min, with a final 10 min hold. The FID detector operated at 300°C. Empty vials with no rectal glands were injected as blanks after every 10 sample injections and at the beginning and end of each analysis sequence. The samples from each of the major and minor sets were run in four batches, with the contents of each batch determined according to the four generations scored (i.e., first generation collected in the first batch, etc). Twenty two authentic standards were also used.

### GC-FID data analysis

Data from the blank samples were subtracted from the chromatograms to remove peaks not originating from the rectal glands. Peaks retained from all chromatograms were manually checked and corrected for retention time shift, following automatic alignment with the GCalignR package in R (version 1.0.2 [114]). The maximum accepted linear shift (max_linear_shift) in a peak’s retention time (Rt) was 0.05 min, and the maximum and minimum accepted Rt difference from the peak mean (max_diff_peak2mean and min_diff_peak2mean, respectively) was 0.03 min in both. Peak areas were calculated using ACD/Spectrus Processor software version 2019.2.0 (Advanced Chemistry Development Inc., Canada) and the values for different batches were normalised to correct for (minor) batch differences in total peak areas. These values were used as indicators of relative abundances because internal standards were not included in all runs. Only peaks which were detected in at least 50% of samples in at least one sex/mating history category in at least one line in the respective data set were retained for further analysis.

All statistical analyses of peak areas were performed in R Studio (version 1.3.1093 [115]).

Quantitative analyses to test for differences between S06 and isofemale lines in individual peaks were carried out using linear modelling (lm function, tidyverse package, version 1.3.0 [116]). Following the approaches of Hoffmann and Parsons [117], David et al. [118] and several other studies (e.g. [83, 89, 90]), significant F ratios for the variation between lines as compared to between replicate variation, both within and between generations, indicates heritable differences between the lines compared. For each peak showing significant line variation, we then investigated the contributions of the different lines to the overall difference by calculating the estimated marginal mean for each line, together with its confidence limits, using the emmeans package [119]. All these analyses were carried out after transforming peak area values appropriately to meet the assumptions of normality and homoscedacity required. The BestNormalize package was used to select the appropriate transformation for each peak [120]. OrderNorm, which normalises the ranks of the response variable to a standard normal distribution (i.e., zero mean and unit variance [121]) was selected in almost all cases, including all those where significant differences were found. Emmeans values were back-transformed for display purposes in Figs 1 and 2 using the predict function in the *stats* package [115, 122]. Significant differences in all analyses were identified after false discovery rate (FDR) adjustment of p values (p.adjust function, tidyverse package [116]).

To test for overall line and locality effects, we first performed PERMANOVAs (adonis function, vegan package [123] on all GC-FID peaks against line effects for each of the three Rt ranges. If line effect was significant, we then ran a PERMANOVA on the data for the respective Rt range against locality, with line nested within the locality effect. To visualise locality differences revealed in the latter analyses, we first performed principal component analyses (PCAs, using the prcomp function) of the GC-FID peaks to obtain a reduced number of variables (fewer than the sample size for each locality). Then linear discriminant analysis (LDA) was conducted on those principal components against locality (lda function, MASS package [124]).

Pairwise Spearman correlations were computed for all pairs of peaks showing significant line differences across all lines in the major set virgin males (with and without the S06 data) and all correlation values were tested for statistically significant differences from zero after FDR adjustment of p values as above.

### SPME GC-MS methods

Sample preparation for the SPME GC-MS analysis was as per the GC-FID analysis above except that the dissected glands were placed in 10 ml screw-cap SPME headspace vials (Sigma Aldrich) before being stored at -80°C until analysed. As per Castro-Vargas et al. [47], the SPME vials had been pre-incubated at -400°C over the previous night to remove potential volatile contaminants. Three replicates from different cages of the S06 strain (~ generation 128) were analysed for each sex/mating history combination.

The GC-MS methods followed those of Castro-Vargas et al. [47], again utilising a Shimadzu TQ8050 gas chromatograph (Shimadzu Corporation) fitted with a fused silica DB5 column (30 m x 0.250 mm i.d. x 1 μm film thickness; Agilent Technologies). Briefly, each sample was preincubated for 5 min at 30°C before a 30 min adsorption into a conditioned polydimethylsiloxane (PDMS) SPME fibre (30 μm thickness; Restek Corporation, PA, USA) at 50°C with constant agitation at 750 rpm. Samples were then desorbed at 250°C for 60 s in an injector fitted with a Topaz inlet liner (Restek Corporation) in a splitless injection mode. The carrier gas was helium, at a total flow rate of 1 mL/min. An initial 40°C oven temperature was held for 1 min and then increased, firstly to 100°C at 10°C/min with a 3 min hold, then to 325°C at 30°C/min with a 10 min hold. The ion source was held at 230°C and detection was set for m/z values from 20 to 600, with a scan interval of 0.3 s. Blank controls were used as per the GC-FID methods above.

All chromatograms were analysed with ACD/Spectrus Processor software version 2019.2.0 (Advanced Chemistry Development Inc., Canada). Blank chromatograms were subtracted from sample chromatograms and peaks were manually aligned to correct for retention time drift. Peak areas were calculated as above and quantitative analysis was performed on peak areas for every peak that was present in at least 50% of the samples in at least one of the four sex/mating history categories. Thirty seven of the peaks had previously been identified by matching equivalent extracts against authentic standards [47, 57, 61, 125]. Other peaks were tentatively identified by detailed analysis of their spectra and comparison of their spectra with the NIST libraries of known compounds with similar KIs (MS Search version 2.3).

## Supporting information

S1 Fig Illustrative GC-FID chromatograms. **Part A** shows a complete chromatogram from a mixed S06 male sample (upper) and a segmented version of the same chromatogram (lower) in which the Y axes of certain segments have been adjusted to facilitate visualisation of smaller peaks. The 15 ‘calibrator’ peaks used in the cross referencing to GC-MS data (see text) are labelled, with abbreviations as per S3 Table. **Part B** shows segmented, Y axes-adjusted, chromatograms of three samples in which the 26 significantly varying peaks are labelled according to their Rt values and, where known, the abbreviations for their names from S3 Table. The upper, middle and lower samples are from NB28, MB64 and S06 mixed males, respectively.
(DOCX)

S2 Fig Rt-KI calibration curve for imputing Ki values for GC-FID peaks. Observed KIs from the GC-MS analysis for the 15 “calibrator” peaks (see S1 Fig) whose identities had been determined with both technologies were plotted against the Rts for those compounds in the GC-FID analyses. Linear relationships were evident for peaks up to Rt 13.59 (green), and from Rt 17.37 onwards (blue) and a multinomial function was fitted for the intervening region (red). Best fit equations for the three relationships are also shown. KI values for all the other GC-FID peaks were imputed by interpolation between the values for adjacent peaks with known KIs in the two linear ranges and using the polynomial function indicated for the intervening region.
(DOCX)

S3 Fig Mass spectra and, where identified, names of the 19 GC-MS peaks matched with GC-FID peaks in S06 males in S4 Table. Peaks are labelled according to their GC-FID Rts and imputed KIs. Names for the five compounds identified against authentic standards are given without asterisks while the five that were tentatively identified by reference to the NIST database are indicated with an asterisk. Also shown are extracts of the gas chromatograms from the GC-MS analysis showing the 19 peaks. Rts on those chromatograms do not match those in the GC-FID analyses because of differences in the chromatographic procedures.
(DOCX)

S1 Table Linear modelling results for peaks in each of the five data sets and imputed KI values for all peaks. F values from the modelling are shown and significance after FDR correction is indicated with asterisks. * p < 0.05, * p < 0.01, * p < 0.001. A dash indicates the peak was not detected in that data set. Imputed KI values were determined from the calibration curve in S2 Fig.
(DOCX)

S2 Table Emmeans for OrderNorm transformed peak areas, plus their 95% confidence limits, for peaks showing significant variation between lines. **Panel A** shows the results for virgin males from the major set of lines, **Panel B** shows the results for mixed males from the major set and **Panel C** the results for mixed males from the minor set of lines. As per Table 2, peaks are bolded, underlined or neither if they are classified as major, intermediate or minor in abundance. Their sex bias (also taken from Table 2) in samples from single/mixed sex cohorts is indicated as follows: Msp = male specific, Mse = male selective, Fse = female specific, Fse = female selective, nd = not detected and ~ = not sex biased, and virgin and mixed sex mating history categories are indicated before and after the slash, respectively. FDR-corrected significance values for F statistics are indicated with asterisks (* p < 0.05, ** p < 0.01, *** p< 0.001). The OrderNorm transformation used to ensure normality and homoscedacity also scaled the values to zero mean and unit variance. Confidence limits are given in parentheses. Letter codes are used to show the results of *post hoc* pairwise contrasts between lines, where lines with the same letters are not significantly different from one another. Note that peaks 4.17 and 8.06 in Panel C do not appear in Table 2 because they did not meet the inclusion criterion (detected in > 50% of samples in at least one sex/mating status category in at least one line) for analysis in the major set of lines and therefore were not classified for abundance or sex bias.
(DOCX)

S3 Table Thirty seven previously identified rectal gland GC-MS peaks in Castro-Vargas et al.^⁕^. KIs given in that study and abbreviations used in S1 and S3 Figs are also shown. Four additional compounds tentatively identified by GC-MS in the current study were 2-methyl 3-hexanol, *n*-butylcyclopentane and two isomers of borneol (2M3H, NBCP, Bor1 and Bor2, respectively).
(DOCX)

S4 Table Alignment of GC-FID and GC-MS profiles in S06 males. For each of the two technologies, data are given for KI, maximum abundance (“Abund.”, i.e., relative abundance in the sex/mating history where it was highest; maj, int and min = major, intermediate and minor, respectively), sex-dependent abundances in virgin and mixed samples (“Sex differences”), and, where known, compound names. Rt values and significant line variation (“sig”) are also indicated for the GC-FID peaks. Peaks are listed in ascending KI order, with the data for calibrator peaks with the two technologies on the same line. Also shown on the same line are 27 pairs of peaks whose imputed GC-FID KI and observed GC-MS KI were no more than four units apart (Best match) and clearly more similar to each other than the immediately adjacent peaks (Next before and Next after). These 27 pairs were then further filtered in respect of their sex-dependent abundances in virgin and mixed samples (before or after the comma respectively, where M or F = present in all male and female samples, respectively, m or f = present in at least one but not all of the respective samples, nd = not detected, na = not assayed, and > or < represent differences of at least 10 fold in the mean peak areas for the two sexes). Compatibility was rated high (YY) for 15 pairs of peaks whose sex-specific expression in each type of sample was the same with both technologies (whether present in all replicates of each type of sample or not, for peaks 5.20, 6.64, 9.58, 10.05, 11.46, 11.70, 11.93, 11.97, 13.95, 14.37, 18.42) or not assayed with one technology (minor data set peaks 7.08, 8.06, 11.16, 11.50). Compatibility was considered acceptable (Y) for five other pairs showing a presence/absence difference in which the presence involved an m or f in a peak whose maximum abundance was still only minor (peaks 6.21, 13.88), or the presence still showed a greater than ten fold bias towards the sex in question (peaks 6.30, 17.07, 17.17). The remaining seven of the 27 KI-matched pairs were considered incompatible (N, for peaks 4.23, 11.04, 15.38, 15.59, 16.32, 16.94, 17.01) in respect of their sex-dependent abundances in either virgin or mixed samples.
(DOCX)

S1 Dataset Raw peak areas of peaks detected by GC-FID in the minor set of isofemale lines. This Data Set contains the peaks that were each present in at least half the samples in at least one of the four sex/mating history categories. The “Cage” column denotes the replicates for each of the lines in each generation. “Gen” refers to the generation in which the lines were screened. Headings for columns F—DU give the respective retention times (Rts) of the peaks screened and the data in body of the table are peak areas after baseline adjustment and removal of contaminants from the traces.
(CSV)

S2 Dataset Raw peak areas of S06 peaks detected by SPME GC-MS. This Data Set contains all the peaks that were present in at least half the S06 samples in at least one of the sex/mating history categories. Observed KIs are shown across the top and the data in the body of the table are peak areas after baseline adjustment and removal of contaminants from the traces.
(CSV)
