# Supplementary figures and images for: Genetic variation for rectal gland volatiles among recently collected isofemale lines and a domesticated strain of Queensland fruit fly, Bactrocera tryoni (Diptera: Tephritidae)
Source: PLoS One. 2023 Apr 28;18(4):e0285099. doi: 10.1371/journal.pone.0285099 (PMC10146519; doi:10.1371/journal.pone.0285099)

S1 Figure. Illustrative GC-FID chromatograms.


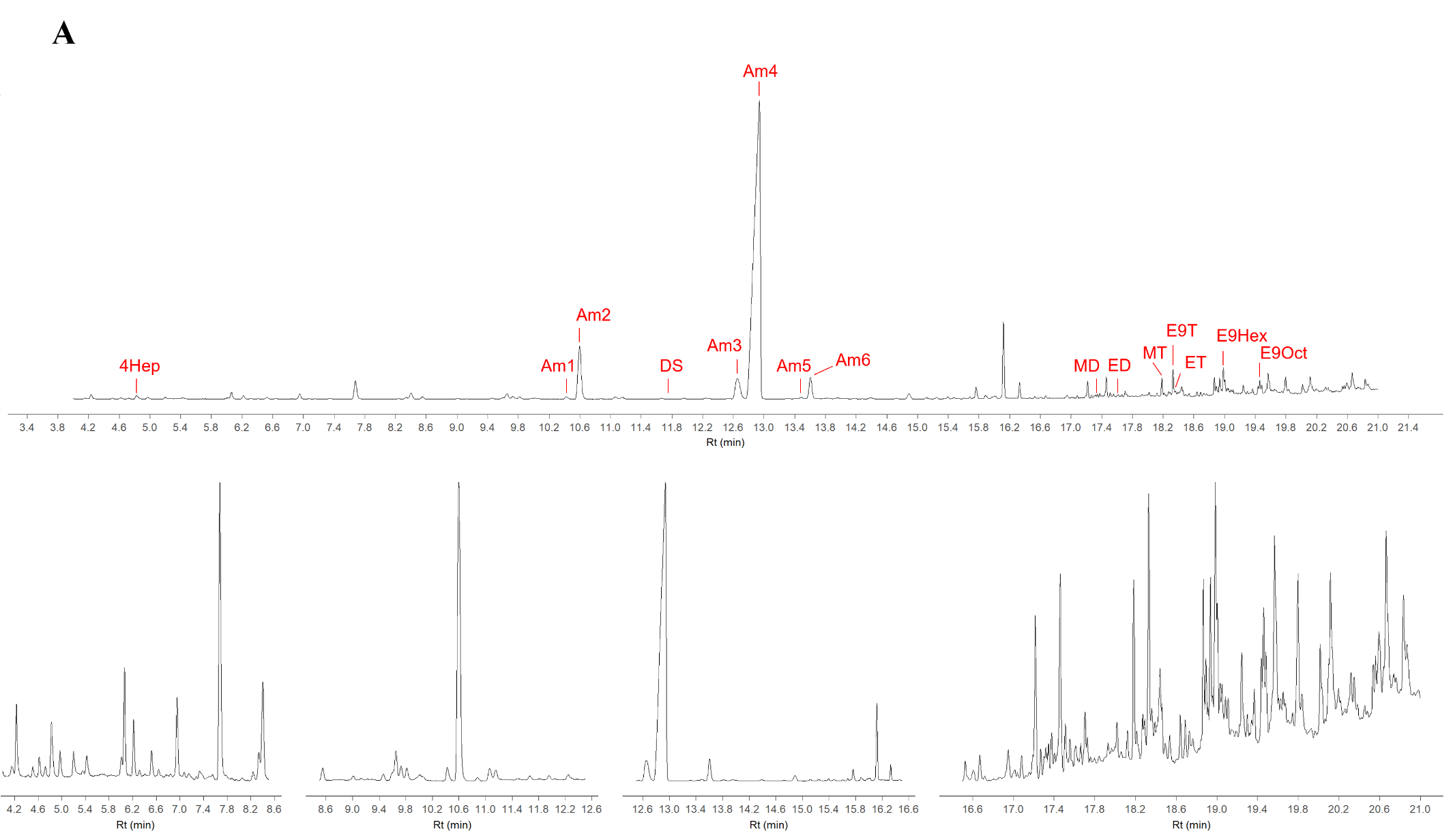


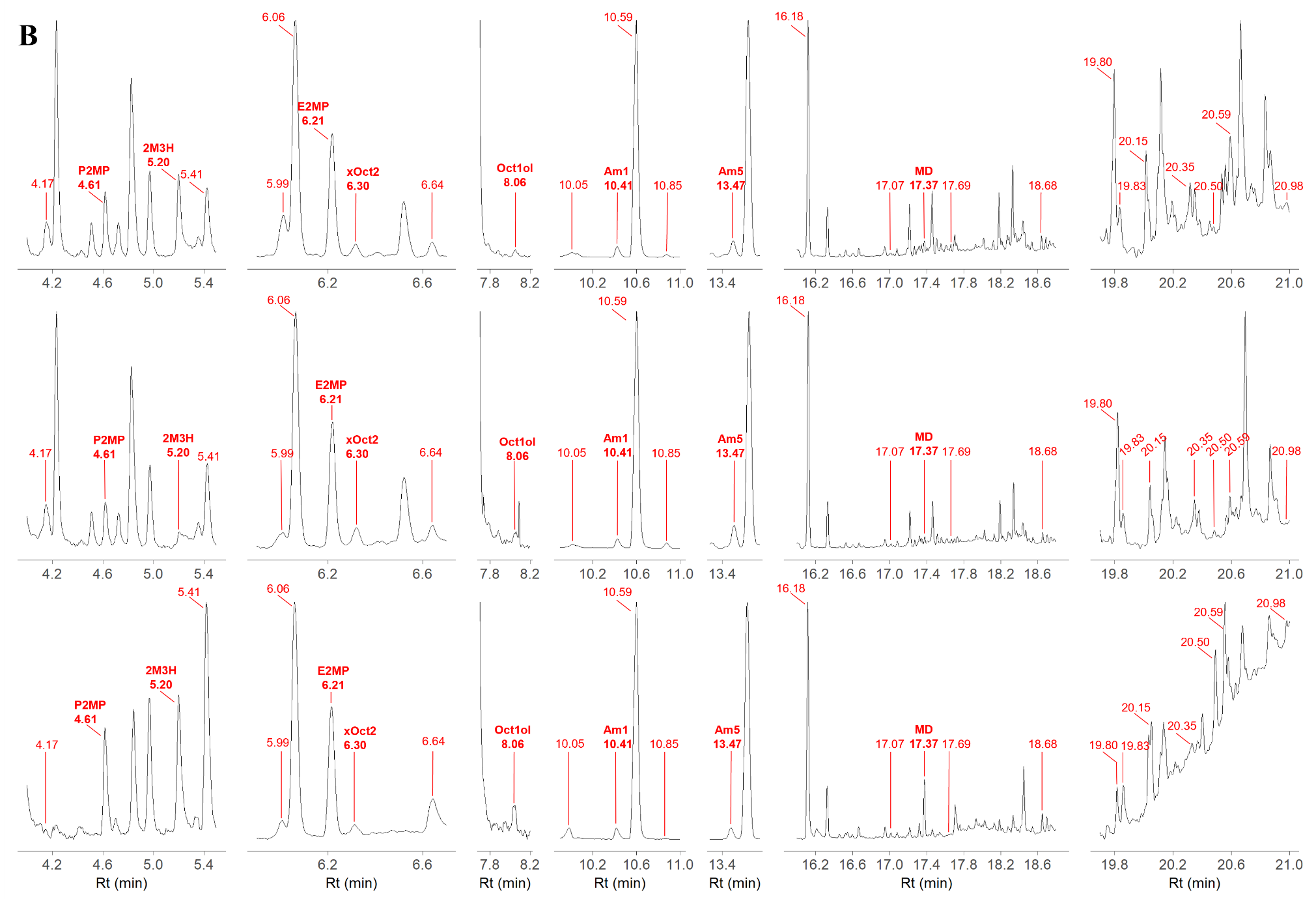

Supplement: S1 Fig — Part A shows a complete chromatogram from a mixed S06 male sample (upper) and a segmented version of the same chromatogram (lower) in which the Y axes of certain segments have been adjusted to facilitate visualisation of smaller peaks. The 15 ‘calibrator’ peaks used in the cross referencing to GC-MS data (see text) are labelled, with abbreviations as per S3 Table. Part B shows segmented, Y axes-adjusted, chromatograms of three samples in which the 26 significantly varying peaks are labelled according to their Rt values and, where known, the abbreviations for their names from S3 Table. The upper, middle and lower samples are from NB28, MB64 and S06 mixed males, respectively. (DOCX) [file pone.0285099.s001.docx]

S2 Figure. Rt-KI calibration curve for imputing Ki values for GC-FID peaks.


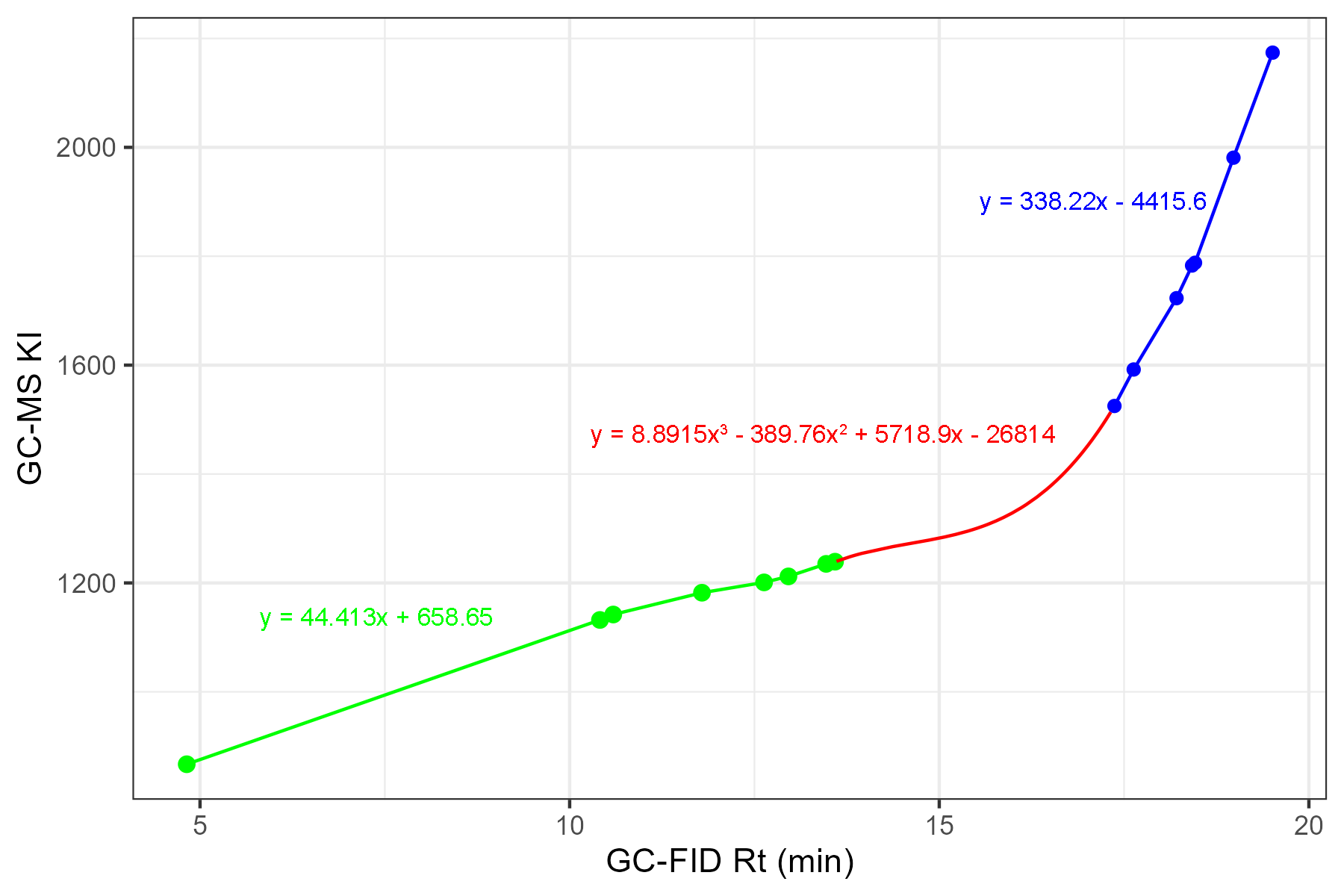

Supplement: S2 Fig — Observed KIs from the GC-MS analysis for the 15 “calibrator” peaks (see S1 Fig) whose identities had been determined with both technologies were plotted against the Rts for those compounds in the GC-FID analyses. Linear relationships were evident for peaks up to Rt 13.59 (green), and from Rt 17.37 onwards (blue) and a multinomial function was fitted for the intervening region (red). Best fit equations for the three relationships are also shown. KI values for all the other GC-FID peaks were imputed by interpolation between the values for adjacent peaks with known KIs in the two linear ranges and using the polynomial function indicated for the intervening region. (DOCX) [file pone.0285099.s002.docx]
