# Supplementary material for: Genetic variation for rectal gland volatiles among recently collected isofemale lines and a domesticated strain of Queensland fruit fly, Bactrocera tryoni (Diptera: Tephritidae)
Source: PLoS One. 2023 Apr 28;18(4):e0285099. doi: 10.1371/journal.pone.0285099 (PMC10146519; doi:10.1371/journal.pone.0285099)
Supplement: S3 Fig — Peaks are labelled according to their GC-FID Rts and imputed KIs. Names for the five compounds identified against authentic standards are given without asterisks while the five that were tentatively identified by reference to the NIST database are indicated with an asterisk. Also shown are extracts of the gas chromatograms from the GC-MS analysis showing the 19 peaks. Rts on those chromatograms do not match those in the GC-FID analyses because of differences in the chromatographic procedures. (DOCX) [file pone.0285099.s003.docx]

S3 Figure. Mass spectra and, where identified, names of the 19 GC-MS peaks matched with GC-FID peaks in S06 males in S4 Table.

| GC-FID peak | SPME GC-MS peak | |
| --- | --- | --- |
|  | Mass spectrum | GC Peak |
| Rt 5.20*  KI 883  2-Methyl 3-hexanol | 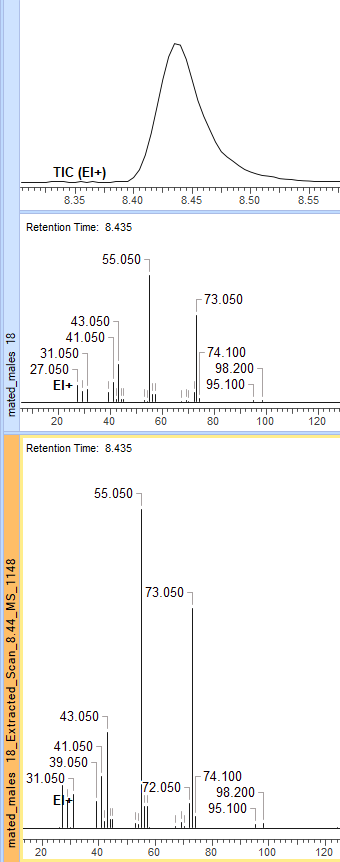 | 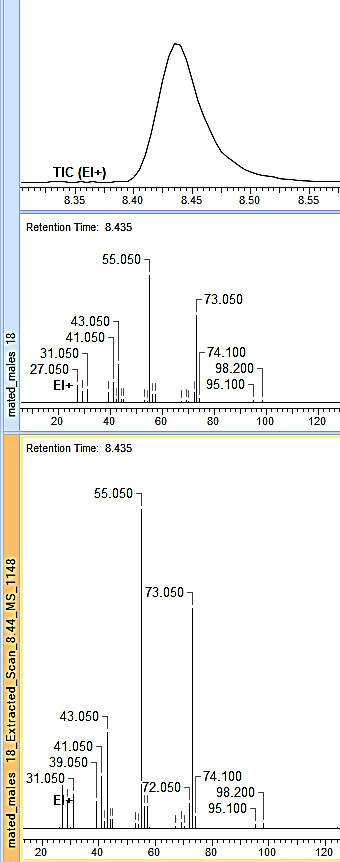 |
| Rt 6.21  KI 933  Ethyl 2-methylpentanoate | 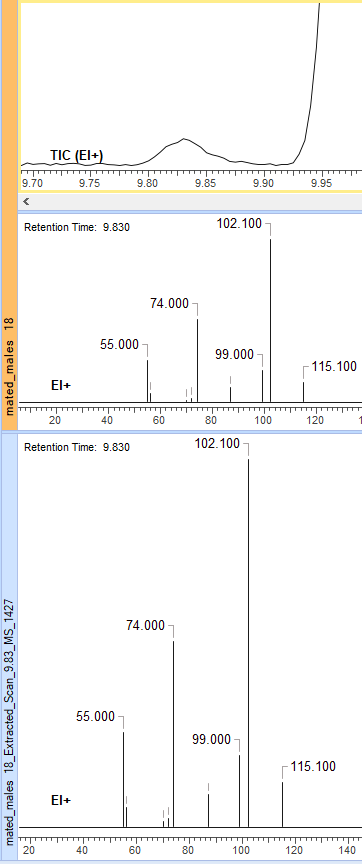 | 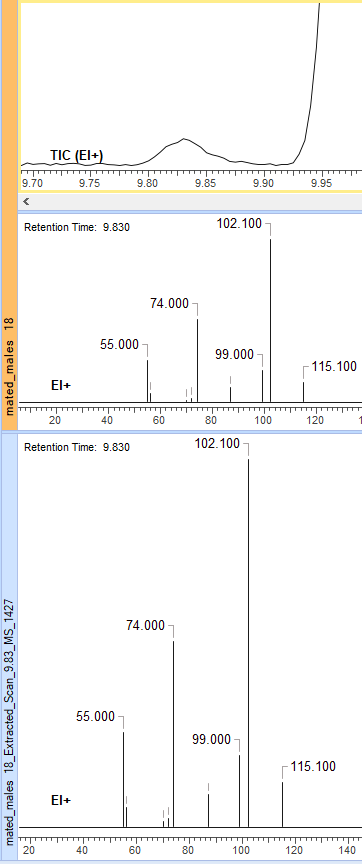 |
| Rt 6.30  KI 939  *x*-Octenal isomer 2 | 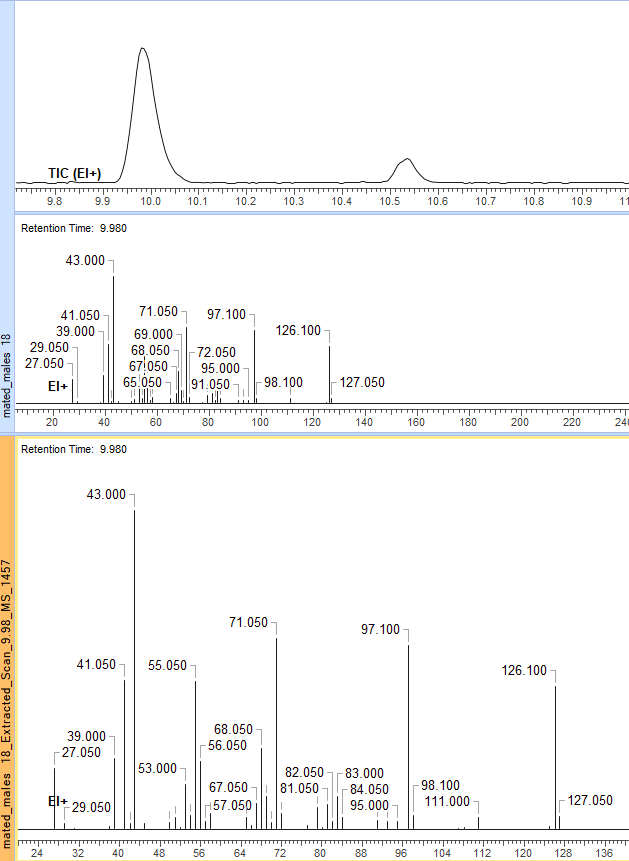 | 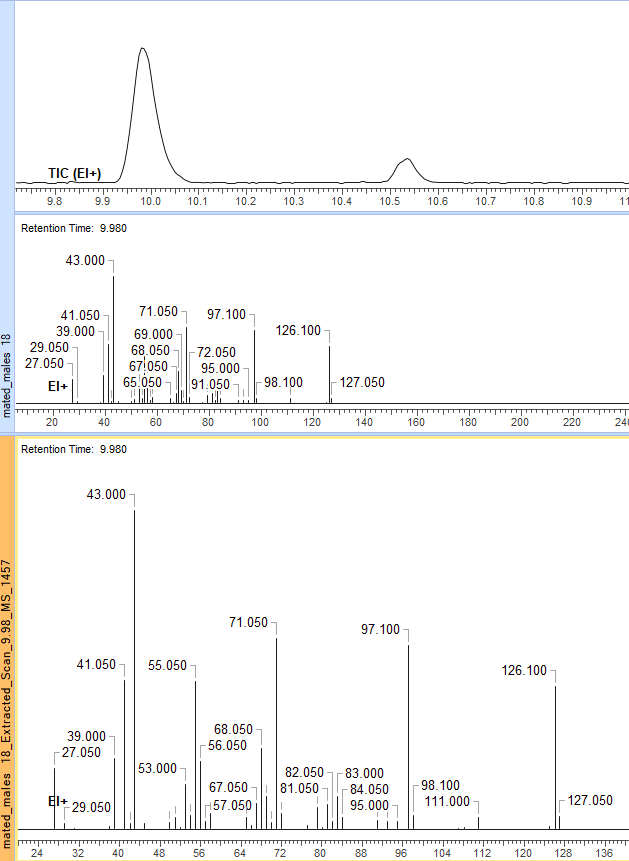 |
| Rt 6.64*  KI 955  *n*-Butyl cyclopentane | 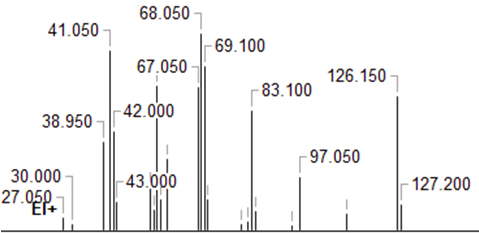 | 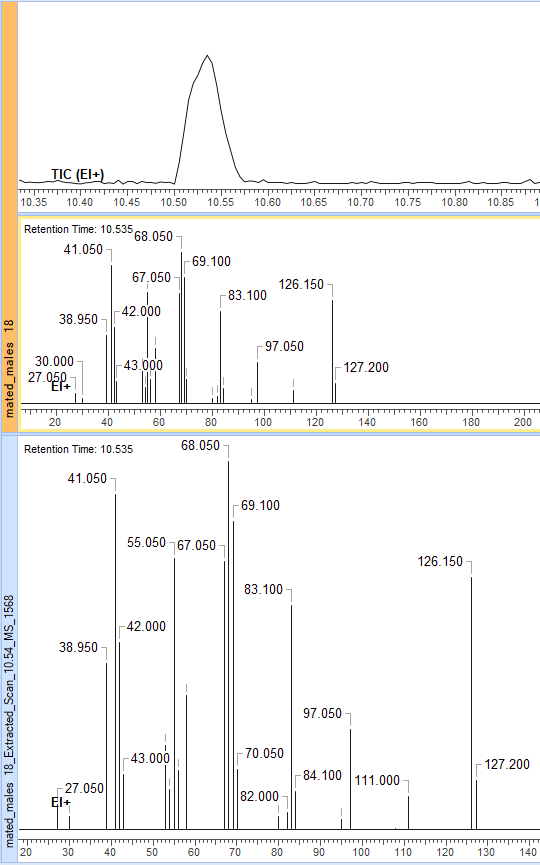 |
| Rt 7.08*  KI 972  Phenol | 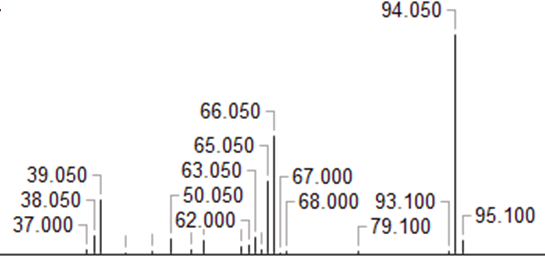 | 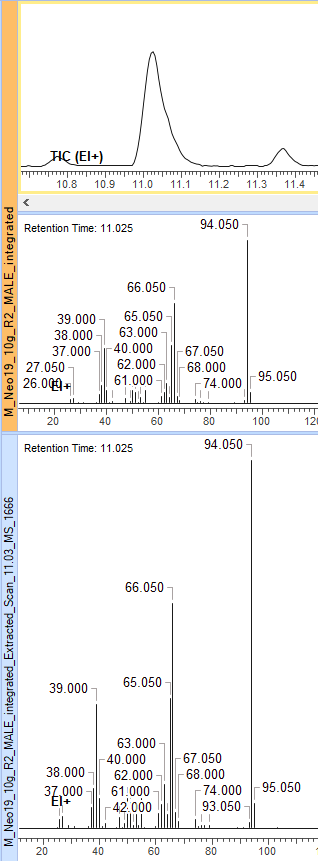 |
| Rt 8.06  KI 1019  *n*-Octen-1-ol | 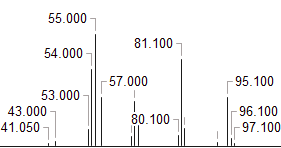 | 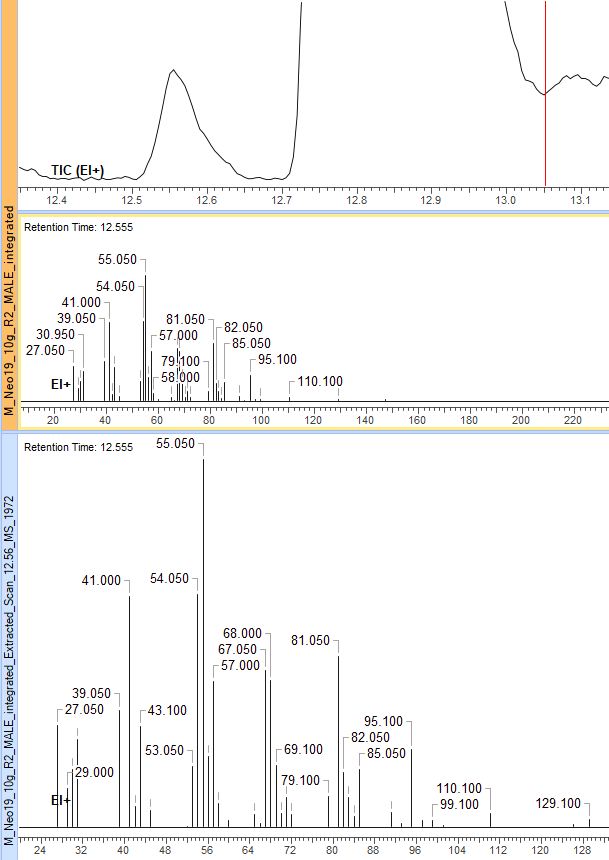 |
| Rt 9.58  KI 1094  *N*-(2-Methylpropyl)  Propanamide | 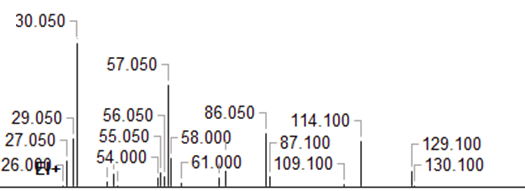 | 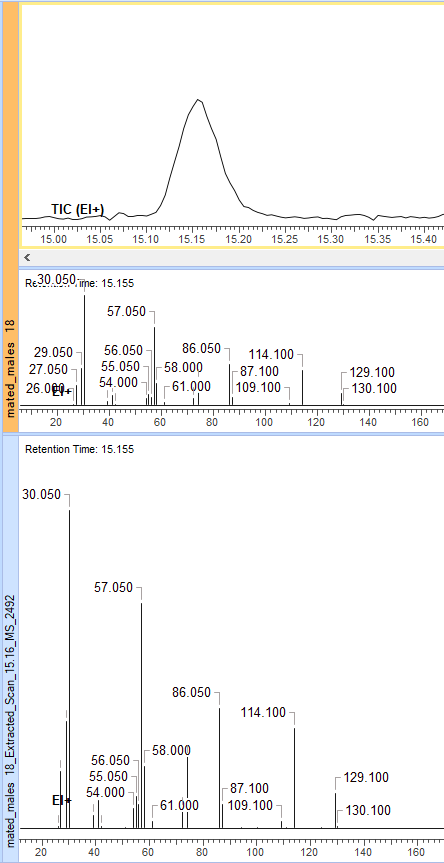 |
| Rt 10.05  KI 1115 | 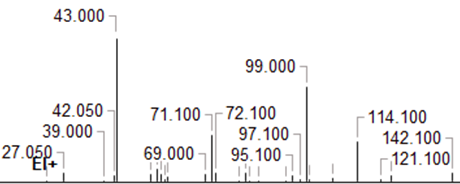 | 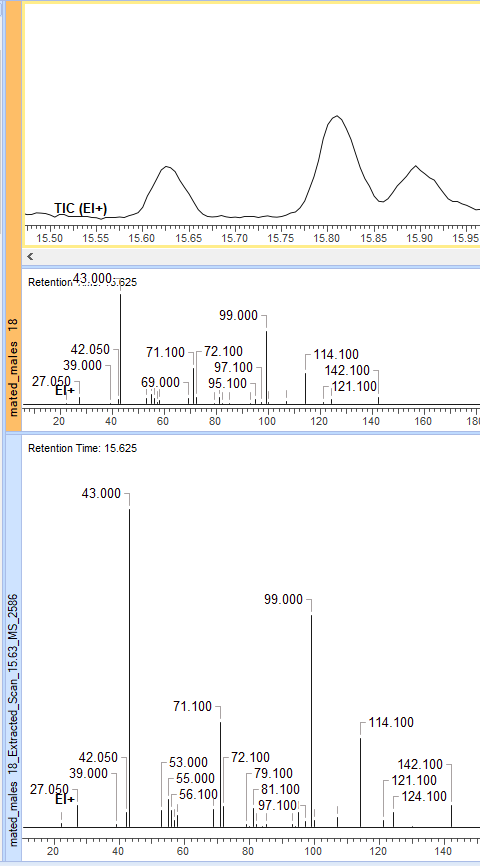 |
| Rt 11.16  KI 1164 | 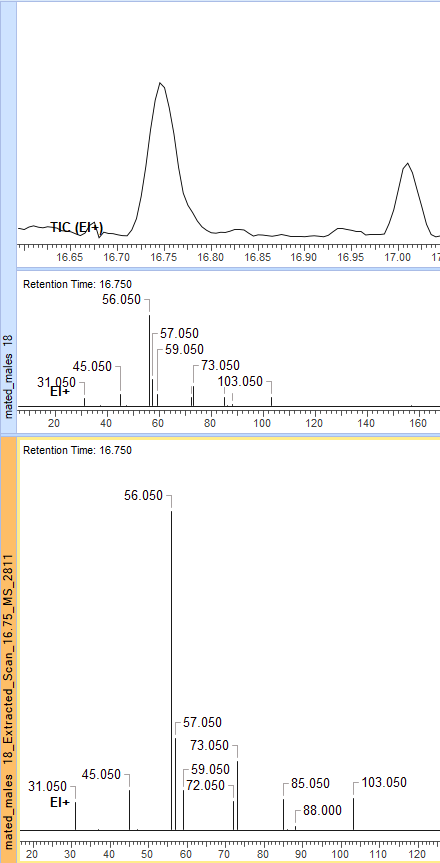 | 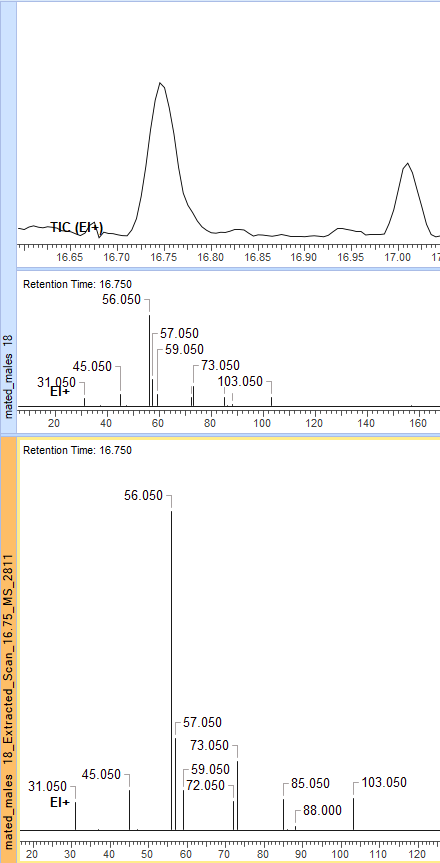 |
| Rt 11.46  KI 1171  2-Bornanone | 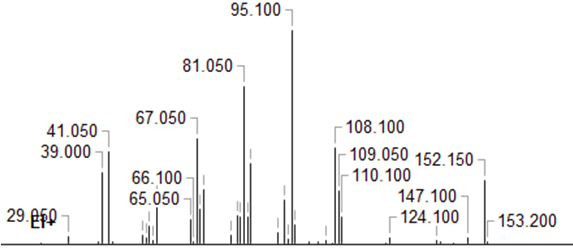 | 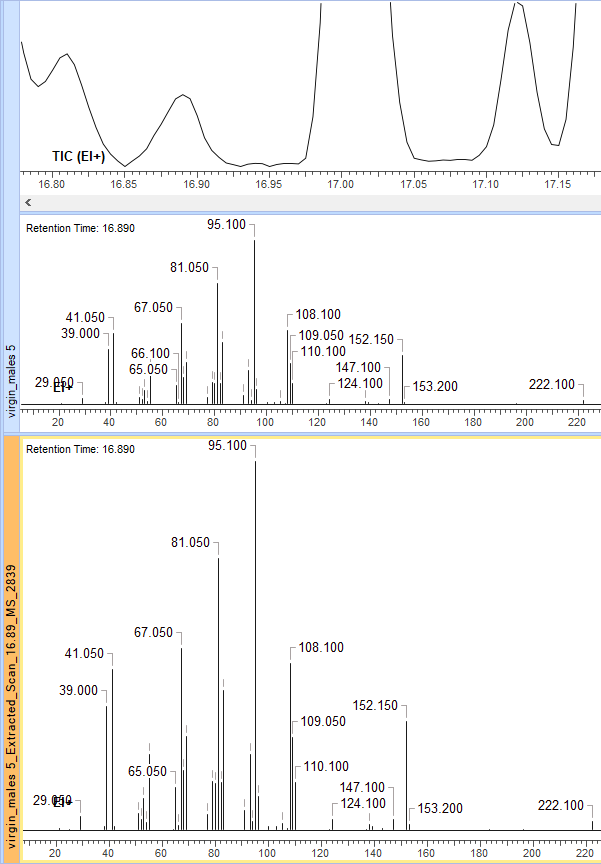 |
| Rt 11.50  KI 1173 | 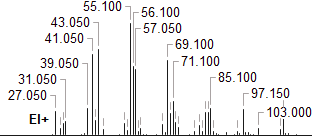 | 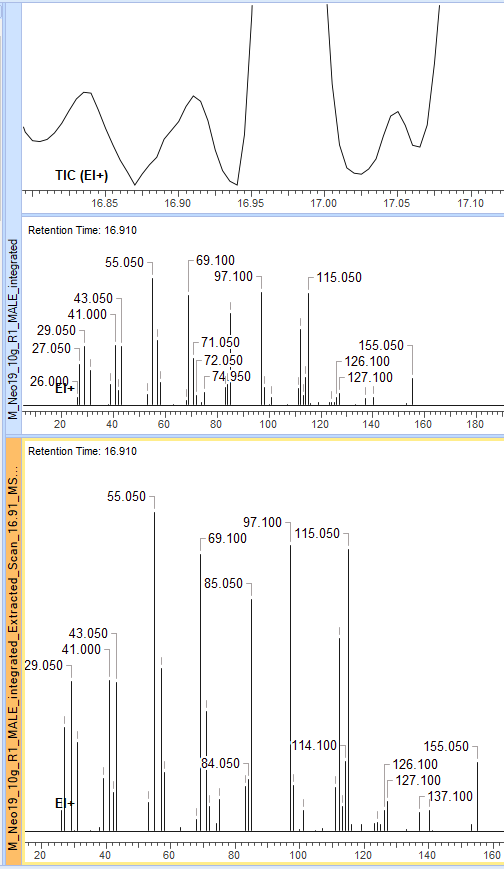 |
| Rt 11.70*  KI 1179  Borneol isomer 1 | 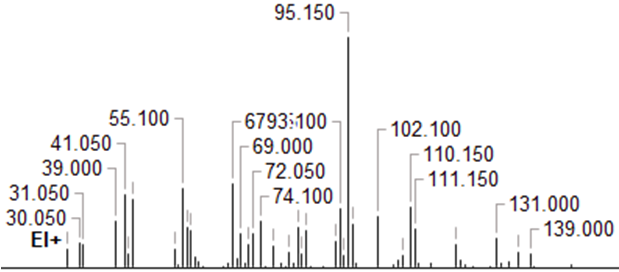 | 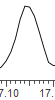 |
| Rt 11.93*  KI 1187  Borneol isomer 2 | 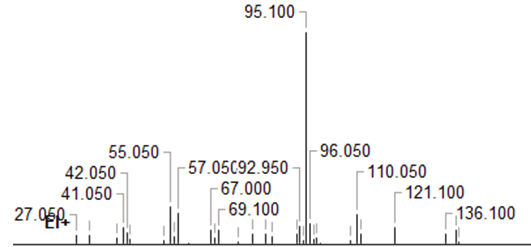 | 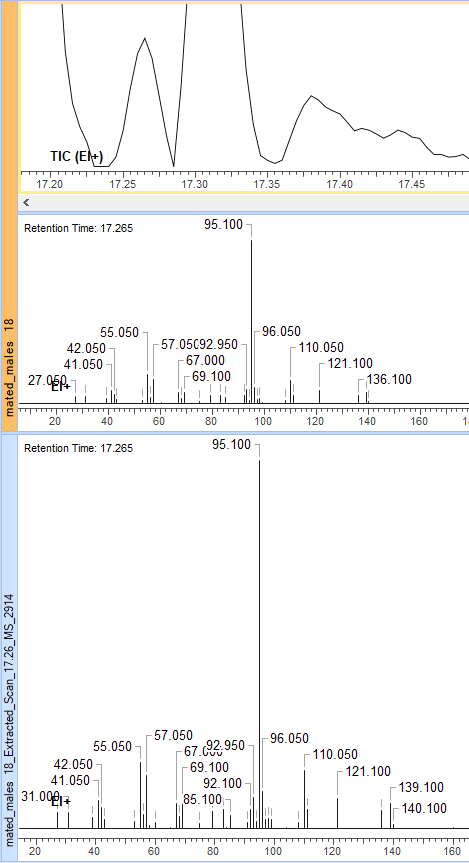 |
| Rt 11.97  KI 1188 | 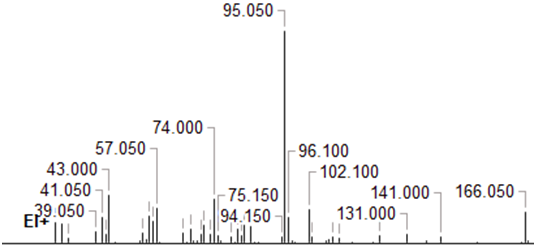 | 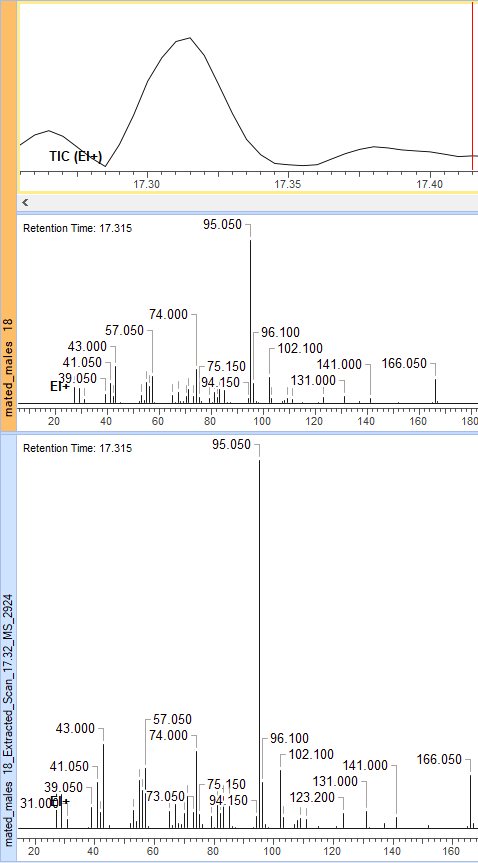 |
| Rt 13.88  KI 1253 | 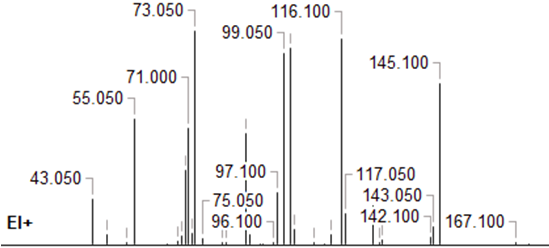 | 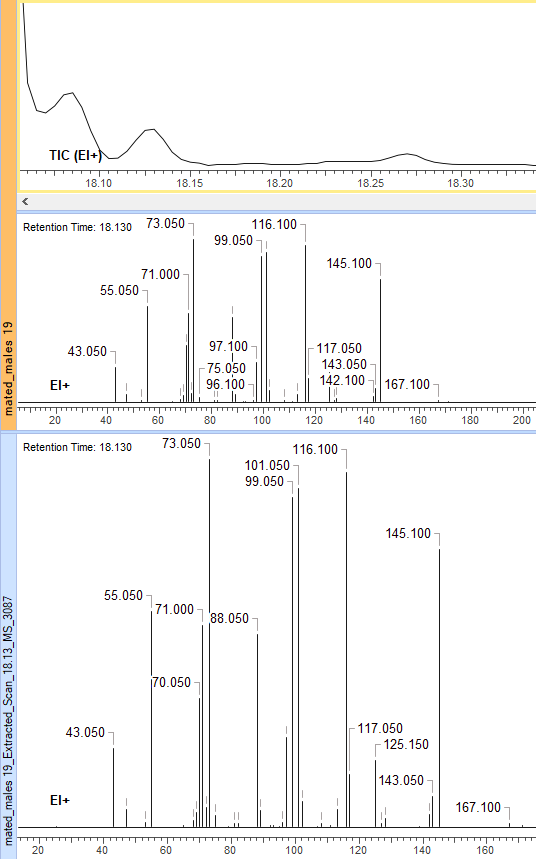 |
| Rt 13.95  KI 1256 | 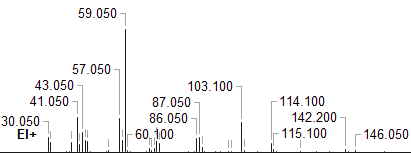 | 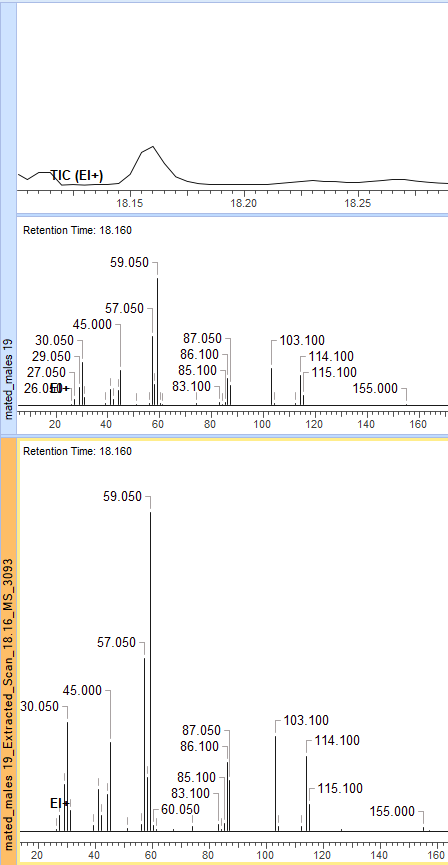 |
| Rt 14.37  KI 1267 | 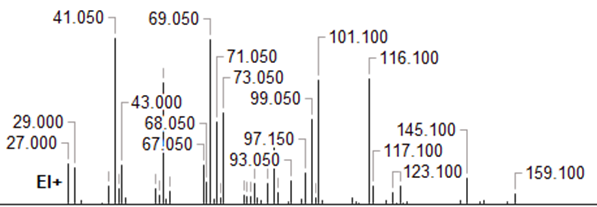 | 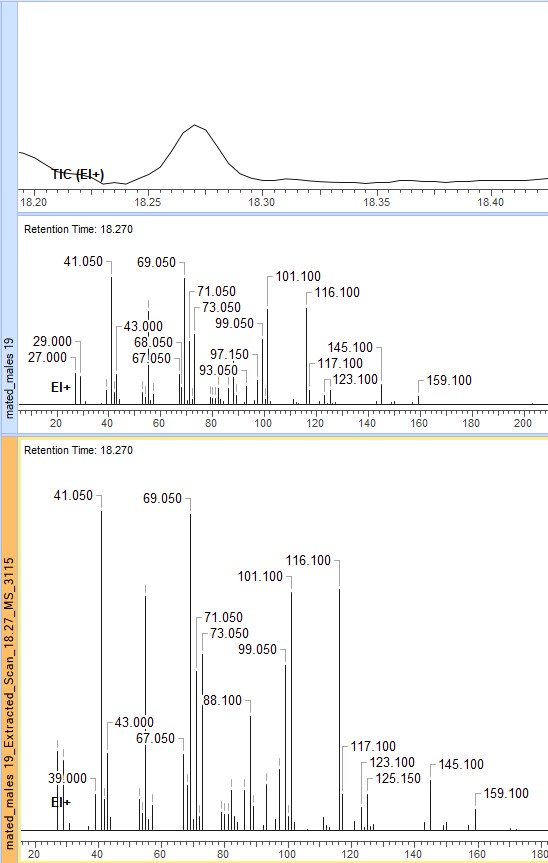 |
| Rt 17.07  KI 1464 | 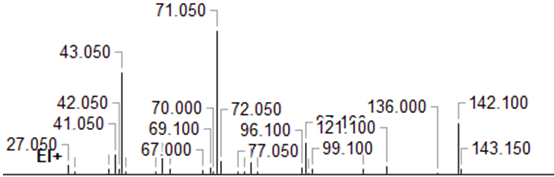 | 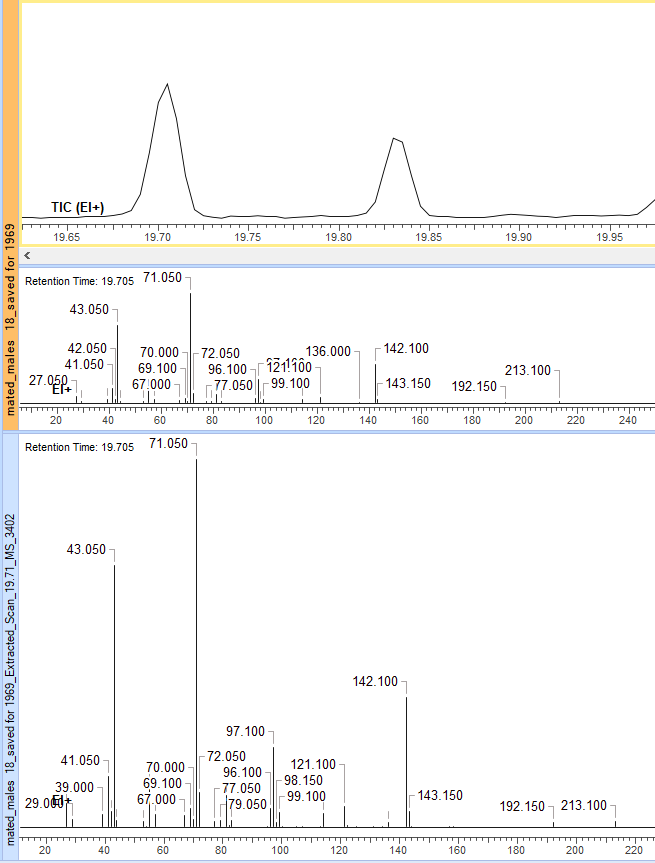 |
| Rt 17.17  KI 1482 | 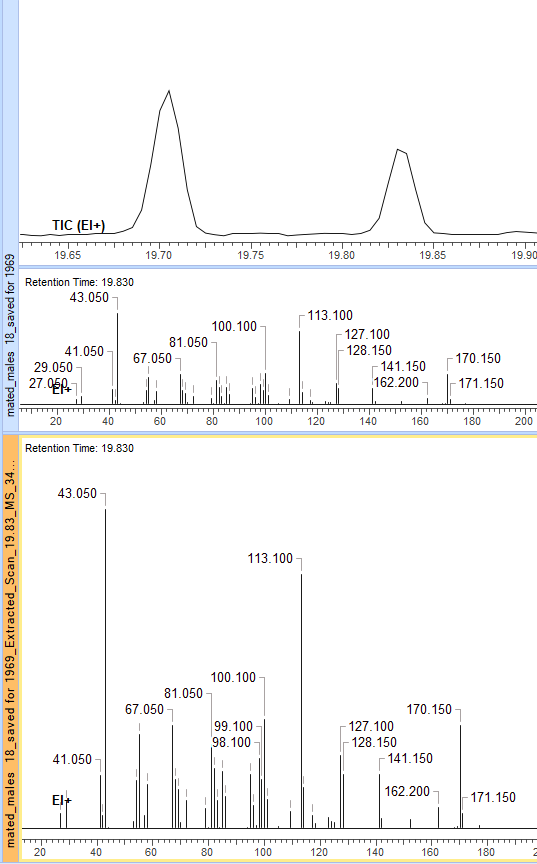 | 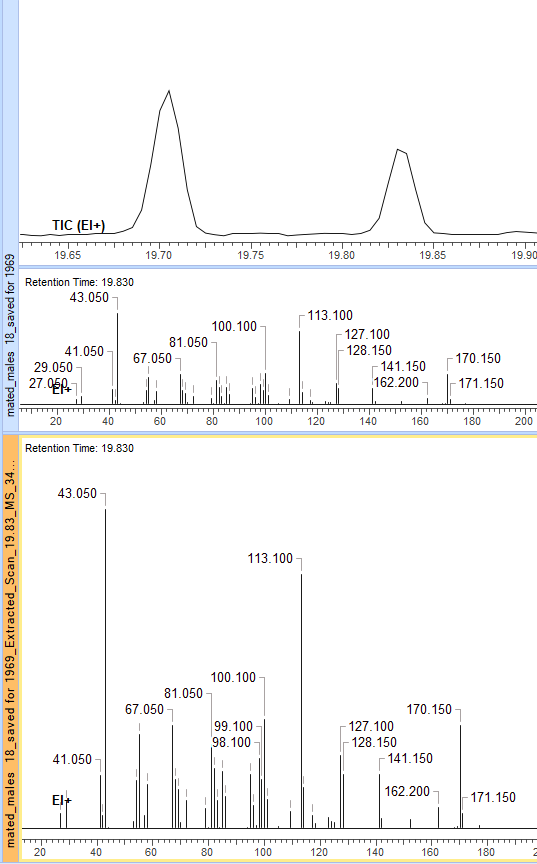 |
