# Supplementary material for: Genetic variation for rectal gland volatiles among recently collected isofemale lines and a domesticated strain of Queensland fruit fly, Bactrocera tryoni (Diptera: Tephritidae)
Source: PLoS One. 2023 Apr 28;18(4):e0285099. doi: 10.1371/journal.pone.0285099 (PMC10146519; doi:10.1371/journal.pone.0285099)
Supplement: S1 Table — F values from the modelling are shown and significance after FDR correction is indicated with asterisks. * p < 0.05, * p < 0.01, * p < 0.001. A dash indicates the peak was not detected in that data set. Imputed KI values were determined from the calibration curve in S2 Fig. (DOCX) [file pone.0285099.s004.docx]

**S1 Table.** Linear modelling results for peaks in each of the five data sets and imputed KI values for all peaks.

|  |  | **Major set** | |  |  | |  | **Minor set** | |
| --- | --- | --- | --- | --- | --- | --- | --- | --- | --- |
| Rt | Imputed KI | Virgin males | | Virgin females | Mixed males | | Mixed females | Mixed males | |
| 4.07 | 831 | - |  | - | 1.59 |  | - | - |  |
| 4.17 | 836 | - |  | - | - |  | - | 2.72 | * |
| 4.23 | 839 | 0.85 |  | 1.07 | 1.07 |  | 1.07 | 0.48 |  |
| 4.61 | 857 | 8.87 | *** | - | 4.01 |  | - | 3.01 | * |
| 4.82 | 867 | 1.12 |  | 1.37 | 8.49 |  | - | 0.62 |  |
| 4.96 | 874 | 1.58 |  | 1.89 | 1.50 |  | - | 1.07 |  |
| 5.20 | 885 | 8.38 | *** | - | 1.23 |  | - | 1.21 |  |
| 5.41 | 895 | 5.10 | ** | - | 4.37 |  | - | 3.18 | * |
| 5.99 | 922 | 3.30 | * | - | 1.35 |  | - | - |  |
| 6.02 | 924 | - |  | - | - |  | - | 1.89 |  |
| 6.06 | 926 | 1.52 |  | 1.62 | 5.56 |  | 1.04 | 3.03 | * |
| 6.21 | 933 | 0.53 |  | 0.33 | 3.44 |  | - | 2.73 | * |
| 6.30 | 937 | 4.97 | ** | - | 3.81 |  | - | 3.05 | * |
| 6.53 | 948 | - |  | - | - |  | - | 1.18 |  |
| 6.64 | 953 | 5.19 | ** | - | 2.22 |  | - | 1.89 |  |
| 6.94 | 967 | 1.27 |  | - | 1.32 |  | - | 0.47 |  |
| 7.08 | 974 | - |  | - | - |  | - | 0.72 |  |
| 7.66 | 1002 | 2.71 |  | 2.73 | 0.39 |  | 1.06 | 1.59 |  |
| 7.79 | 1008 | - |  | - | 2.41 |  | - | - |  |
| 8.06 | 1021 | - |  | - | - |  | - | 2.94 | * |
| 8.38 | 1036 | 1.24 |  | 0.65 | 1.92 |  | 1.38 | 0.77 |  |
| 8.53 | 1043 | 1.05 |  | - | 1.53 |  | - | - |  |
| 8.73 | 1052 | - |  | - | 0.92 |  | - | - |  |
| 8.82 | 1057 | - |  | 0.92 | - |  | 4.82 | - |  |
| 9.43 | 1086 | 2.9 |  | - | 3.38 |  | - | - |  |
| 9.58 | 1093 | 1.6 |  | - | 6.25 |  | - | - |  |
| 9.63 | 1095 | - |  | 1.11 | - |  | - | 1.59 |  |
| 9.74 | 1100 | - |  | - | - |  | - | 2.17 |  |
| 9.79 | 1103 | - |  | - | 0.96 |  | - | - |  |
| 9.98 | 1112 | - |  | - | 0.78 |  | - | - |  |
| 10.05 | 1115 | 4.66 | ** | - | - |  | - | - |  |
| 10.41 | 1132 | 2.14 |  | 2.89 | 2.38 |  | - | 2.54 | * |
| 10.59 | 1142 | 1.85 |  | 3.23 | 2.18 |  | 5.35 | - |  |
| 10.61 | 1143 | - |  | - | - |  | - | 2.43 |  |
| 10.85 | 1151 | 3.40 | * | 1.36 | 0.80 |  | 1.62 | 3.18 | * |
| 11.04 | 1157 | 0.69 |  | 1.8 | 2.37 |  | - | 0.83 |  |
| 11.16 | 1161 | - |  | - | - |  | - | 0.27 |  |
| 11.27 | 1165 | - |  | 3.66 | - |  | 0.76 | - |  |
| 11.46 | 1171 | 2.73 |  | - | 0.51 |  | - | - |  |
| 11.50 | 1172 | - |  | - | - |  | - | 1.50 |  |
| 11.7 | 1179 | - |  | - | 0.34 |  | - | - |  |
| 11.79 | 1182 | - |  | - | 2.32 |  | - | - |  |
| 11.93 | 1185 | 2.72 |  | - | 1.59 |  | - | - |  |
| 11.97 | 1186 | - |  | - | 1.99 |  | - | - |  |
| 12.27 | 1193 | 0.98 |  | 1.82 | 0.78 |  | - | 1.66 |  |
| 12.45 | 1197 | - |  | - | 6.57 |  | - | - |  |
| 12.56 | 1199 | - |  | - | - |  | - | 1.25 |  |
| 12.63 | 1201 | 0.80 |  | 0.97 | 4.46 |  | 6.5 | 1.45 |  |
| 12.96 | 1211 | 2.37 |  | 0.96 | 4.91 |  | 4.64 | 1.57 |  |
| 13.33 | 1227 | 0.63 |  | - | 0.41 |  | - | - |  |
| 13.47 | 1235 | 5.50 | ** | 1.78 | 3.77 |  | 1.60 | 1.75 |  |
| 13.59 | 1239 | 3.00 |  | - | 5.49 |  | - | 2.48 |  |
| 13.79 | 1248 | 3.01 |  | - | 3.99 |  | - | - |  |
| 13.82 | 1249 | - |  | - | - |  | - | 0.86 |  |
| 13.88 | 1252 | - |  | - | 2.71 |  | - | - |  |
| 13.95 | 1254 | 0.94 |  | - | 1.86 |  | - | 0.76 |  |
| 14.37 | 1276 | 0.73 |  | 1.07 | 2.18 |  | 0.88 | 0.81 |  |
| 14.54 | 1271 | - |  | - | 7.57 |  | - | - |  |
| 14.86 | 1279 | 2.01 |  | 1.17 | 4.37 |  | 1.03 | 0.52 |  |
| 15.07 | 1248 | - |  | - | 1.74 |  | - | - |  |
| 15.22 | 1289 | 1.21 |  | - | 2.42 |  | - | - |  |
| 15.38 | 1295 | 2.9 |  | - | 0.74 |  | - | 0.96 |  |
| 15.45 | 1298 | - |  | - | 0.54 |  | - | 1.02 |  |
| 15.51 | 1300 | - |  | 1.55 | - |  | 0.69 | - |  |
| 15.59 | 1304 | 2.12 |  | - | 9.96 |  | 3.63 | - |  |
| 15.63 | 1306 | - |  | 0.87 | 5.11 |  | - | 0.73 |  |
| 15.67 | 1308 | 1.03 |  | - | 2.32 |  | - |  |  |
| 15.77 | 1314 | 0.79 |  | 1.87 | 3.52 |  | 0.97 | 0.53 |  |
| 15.87 | 1320 | 1.49 |  | 1.13 | 1.79 |  | - | 0.79 |  |
| 15.98 | 1328 | 1.34 |  | - | 1.87 |  | - | - |  |
| 15.99 | 1329 | 0.72 |  | - | 0.87 |  | 0.52 | 0.63 |  |
| 16.11 | 1338 | 1.59 |  | 0.69 | 4.43 |  | - | 0.95 |  |
| 16.14 | 1341 | - |  | - | 0.91 |  | - | - |  |
| 16.18 | 1344 | 9.56 | *** | - | 1.18 |  | - | - |  |
| 16.32 | 1358 | 1.29 |  | 0.95 | 3.67 |  | 0.37 | 0.50 |  |
| 16.45 | 1372 | 2.67 |  | 1.01 | 0.99 |  | 1.97 | 1.11 |  |
| 16.50 | 1377 | - |  | - | 1.48 |  | 1.29 | - |  |
| 16.52 | 1380 | 1.55 |  | - | 1.07 |  | - | 1.15 |  |
| 16.59 | 1389 | 3.03 |  | - | 1.7 |  | - | 0.96 |  |
| 16.66 | 1398 | 0.78 |  | - | 4.92 |  | - | 1.30 |  |
| 16.70 | 1403 | - |  | - | 1.72 |  | - | - |  |
| 16.73 | 1408 | 0.48 |  | - | - |  | - | 0.79 |  |
| 16.78 | 1415 | - |  | - | 1.06 |  | - | - |  |
| 16.85 | 1426 | 1.35 |  | - | 2.39 |  | - | 0.98 |  |
| 16.89 | 1432 | 2.08 |  | - | 1.44 |  | - | - |  |
| 16.94 | 1440 | 1.62 |  | - | 3 |  | - | 0.74 |  |
| 17.01 | 1452 | 2.96 |  | - | 4.23 |  | - | 0.95 |  |
| 17.07 | 1463 | 3.18 | * | - | 0.75 |  | - | 0.76 |  |
| 17.15 | 1478 | 1.34 |  | 0.88 | 0.82 |  | - | - |  |
| 17.17 | 1482 | 0.56 |  | - | 0.9 |  | - | 0.79 |  |
| 17.21 | 1490 | 1.26 |  | 2.24 | 0.62 |  | 1.03 | 0.94 |  |
| 17.26 | 1501 | 1.46 |  | - | 0.65 |  | - | 0.26 |  |
| 17.32 | 1514 | 1.68 |  | 1.68 | 0.56 |  | - | 0.53 |  |
| 17.37 | 1525 | 5.73 | ** | 0.94 | 10.67 |  | 0.66 | 2.37 |  |
| 17.41 | 1535 | 0.65 |  | - | 0.56 |  | - | 0.67 |  |
| 17.45 | 1546 | 1.95 |  | 0.24 | 4.42 |  | 1.06 | 0.55 |  |
| 17.47 | 1551 | - |  | - | - |  | - | 0.48 |  |
| 17.52 | 1564 | 1.22 |  | 1.17 | 1.97 |  | - | 0.72 |  |
| 17.55 | 1571 | 1.53 |  | 1.82 | 1.48 |  | 0.29 | 0.48 |  |
| 17.63 | 1592 | 1.83 |  | 0.77 | 3.31 |  | 0.91 | - |  |
| 17.64 | 1594 | 1.12 |  | - | 0.27 |  | - | - |  |
| 17.69 | 1606 | 3.53 | * | 1.14 | 5.24 |  | 1.15 | 0.82 |  |
| 17.70 | 1608 | - |  | - | - |  | - | 1.85 |  |
| 17.72 | 1612 | 0.99 |  | - | - |  | - | 1.67 |  |
| 17.75 | 1619 | 1.06 |  | - | 5.35 |  | - | 0.82 |  |
| 17.84 | 1639 | 2.12 |  | - | 3.24 |  | - | 2.01 |  |
| 17.92 | 1658 | 0.37 |  | 0.55 | 0.38 |  | 1.62 | - |  |
| 17.96 | 1667 | 1.64 |  | 0.88 | 0.46 |  | 2.15 | 1.85 |  |
| 18.01 | 1678 | 2.63 |  | - | 0.69 |  | - | 0.75 |  |
| 18.02 | 1680 | - |  | - | 0.79 |  | - | - |  |
| 18.1 | 1698 | 1.54 |  | 0.83 | 3.75 |  | 0.59 | - |  |
| 18.12 | 1703 | 1.12 |  | 1.06 | 6.24 |  |  | 1.47 |  |
| 18.17 | 1714 | 1.76 |  | 1.14 | 2.05 |  | 0.51 | 1.02 |  |
| 18.21 | 1723 | 0.38 |  | 0.84 | 3.83 |  | 0.21 | 0.58 |  |
| 18.27 | 1740 | 2.25 |  | 1.05 | 1.39 |  | 1.45 | 0.71 |  |
| 18.32 | 1754 | 1.54 |  | 1.32 | 2.34 |  | 0.16 | 0.98 |  |
| 18.37 | 1769 | 1.24 |  | 1.04 | - |  | 2.12 | 0.45 |  |
| 18.39 | 1774 | 0.7 |  | - | - |  | 0.46 | 0.44 |  |
| 18.40 | 1777 | - |  | 0.87 | - |  | 3.08 | - |  |
| 18.42 | 1783 | 0.83 |  | 1.72 | 7.91 |  | 1.46 | 1.25 |  |
| 18.46 | 1788 | - |  | 1.79 | - |  | 0.48 | - |  |
| 18.48 | 1795 | 1.41 |  | - | 7.23 |  | - | 0.37 |  |
| 18.53 | 1814 | 1.98 |  | 1.51 | 3.73 |  | - | 0.17 |  |
| 18.61 | 1844 | - |  | 1.15 | 0.94 |  | 2.49 | - |  |
| 18.64 | 1855 | 2.39 |  | 0.88 | 1.17 |  | 1.4 | 0.56 |  |
| 18.68 | 1880 | 1.57 |  | - | 26.71 | * | - | 1.14 |  |
| 18.72 | 1884 | 1.31 |  | - | 4.06 |  | 0.47 | 1.28 |  |
| 18.73 | 1888 | - |  | 0.71 | - |  | 0.47 | - |  |
| 18.76 | 1899 | 1.67 |  | - | - |  | - | - |  |
| 18.78 | 1907 | - |  | 0.77 | - |  | 0.06 | - |  |
| 18.8 | 1914 | 0.99 |  |  | 2.02 |  | - | - |  |
| 18.83 | 1925 | - |  | 0.31 | - |  | 0.2 | - |  |
| 18.85 | 1993 | - |  | 1.25 | - |  | - | - |  |
| 18.88 | 1944 | 0.53 |  | 2.42 | 10.63 |  | 0.37 | 0.37 |  |
| 18.89 | 1948 | 1.44 |  | - | 4.95 |  | - | 1.61 |  |
| 18.93 | 1962 | 0.65 |  | 1.31 | 5.59 |  | 1.26 | 0.85 |  |
| 18.98 | 1981 | 0.62 |  | 1.43 | 1.27 |  | 0.21 | 0.79 |  |
| 19.01 | 1992 | 2.19 |  | 1.86 | 0.49 |  | 0.57 | 0.51 |  |
| 19.04 | 2003 | 2.24 |  | - | - |  | - | 0.62 |  |
| 19.05 | 2006 | - |  | - | - |  | - | 0.81 |  |
| 19.08 | 2017 | 1.56 |  | - | 1.54 |  | - | 0.42 |  |
| 19.12 | 2032 | 1.49 |  | - | 0.61 |  | - | 0.86 |  |
| 19.14 | 2039 | - |  | 1.24 | 0.75 |  | 1.53 | - |  |
| 19.15 | 2043 | - |  | - | 1.50 |  | - | - |  |
| 19.20 | 2061 | - |  | - | 1.82 |  | - | - |  |
| 19.25 | 2079 | 1.49 |  | 2.4 | 0.44 |  | 0.69 | 1.34 |  |
| 19.29 | 2094 | 1.92 |  | 1.38 | 0.12 |  | 1.8 | 1.8 |  |
| 19.31 | 2101 | - |  | - | 13.27 |  | - | - |  |
| 19.35 | 2116 | 2.37 |  | 1.57 | 0.60 |  | 0.48 | - |  |
| 19.39 | 2130 | 0.87 |  | 0.94 | 2.62 |  | 0.58 | - |  |
| 19.43 | 2145 | 1.29 |  | 1.71 | 1.82 |  | 0.44 | 1.34 |  |
| 19.48 | 2163 | 1.71 |  | 0.65 | 4.00 |  | - | 0.53 |  |
| 19.51 | 2174 | - |  | 1.42 | 4.37 |  | 0.21 | 0.85 |  |
| 19.56 | 2192 | 1.69 |  | 1.97 | 3.95 |  | 0.46 | 1.53 |  |
| 19.59 | 2203 | - |  | 1.53 | - |  | 0.71 | 0.93 |  |
| 19.62 | 2214 | 0.69 |  | - | 2.45 |  | - | 1.1 |  |
| 19.65 | 2225 | 2.11 |  | - | - |  | - | - |  |
| 19.66 | 2229 | 0.73 |  | 2.09 | 4.81 |  | 5.29 | 1.18 |  |
| 19.70 | 2243 | - |  | 1.66 | - |  | - | - |  |
| 19.72 | 2250 | 1.48 |  | - | 0.71 |  | - | - |  |
| 19.76 | 2265 | - |  | 1.00 | 2.89 |  | 0.63 | 0.62 |  |
| 19.78 | 2272 | - |  | - | 0.65 |  | - | - |  |
| 19.80 | 2283 | 0.63 |  | 0.95 | 1.46 |  | 4.97 | 2.61 | * |
| 19.83 | 2290 | 1.51 |  | - | 3.46 |  | - | 2.64 | * |
| 19.86 | 2301 | 2.37 |  | 0.71 | 1.93 |  | 0.55 | - |  |
| 19.92 | 2323 | - |  | - | 0.50 |  | - | - |  |
| 19.94 | 2331 | - |  | - | 0.66 |  | - | - |  |
| 19.97 | 2341 | - |  | - | 2.37 |  | - | - |  |
| 19.99 | 2349 | - |  | 1.42 | - |  | - | - |  |
| 20.01 | 2357 | 2.83 |  | - | 2.03 |  | 2.86 | - |  |
| 20.05 | 2371 | - |  | 1.13 | - |  | 0.39 | 0.19 |  |
| 20.07 | 2378 | 0.88 |  | 0.8 | 0.60 |  | - | 1.18 |  |
| 20.11 | 2392 | - |  | 0.9 | - |  | 1.56 | 0.86 |  |
| 20.13 | 2400 | 0.75 |  | - | 2.75 |  | 2.14 | 1.35 |  |
| 20.15 | 2407 | - |  | 1.6 | 24.05 | * | - | - |  |
| 20.19 | 2422 | 2.46 |  | - | - |  | - | - |  |
| 20.20 | 2425 | - |  | - | - |  | - | 0.70 |  |
| 20.22 | 2433 | - |  | - | 2.61 |  | - | - |  |
| 20.23 | 2436 | - |  | - | 3.72 |  | - | - |  |
| 20.25 | 2443 | - |  | 0.78 | - |  | 1.58 | - |  |
| 20.31 | 2465 | 3.04 |  | 2.11 | - |  | 0.35 | - |  |
| 20.33 | 2473 | 1.78 |  | - | 7.97 |  | - | 2.29 |  |
| 20.35 | 2480 | 3.22 | * | - | - |  | - | - |  |
| 20.37 | 2487 | - |  | - | 4.53 |  | 2.15 | 2.25 |  |
| 20.40 | 2498 | - |  | - | 0.6 |  | - | - |  |
| 20.42 | 2505 | - |  | - | 0.77 |  | - | - |  |
| 20.50 | 2534 | 3.54 | * | 0.42 | 11.08 |  | 1.70 | 1.26 |  |
| 20.52 | 2542 | 0.80 |  | 1.03 | - |  | - | - |  |
| 20.55 | 2553 | - |  | 1.22 | 2.23 |  | 0.88 | 0.66 |  |
| 20.59 | 2567 | 3.80 | * | - | 3.52 |  | - | 0.62 |  |
| 20.62 | 2578 | 1.52 |  | - | - |  | 0.95 | 1.01 |  |
| 20.64 | 2584 | 0.57 |  | - | 0.57 |  | - | 1.49 |  |
| 20.68 | 2600 | - |  | - | 5.99 |  | 3.38 | 1.28 |  |
| 20.81 | 2647 | 1.25 |  | - | - |  | - | - |  |
| 20.84 | 2658 | 0.82 |  | 2.95 | 2.39 |  | 0.79 | 0.83 |  |
| 20.88 | 2673 | - |  | - | 5.52 |  | 6.91 | 2.04 |  |
| 20.90 | 2680 | - |  | - | 1.39 |  | 4.65 | 0.93 |  |
| 20.98 | 2709 | 4.83 | ** | - | 8.75 |  | - | - |  |
