# Supplementary material for: Genetic variation for rectal gland volatiles among recently collected isofemale lines and a domesticated strain of Queensland fruit fly, Bactrocera tryoni (Diptera: Tephritidae)
Source: PLoS One. 2023 Apr 28;18(4):e0285099. doi: 10.1371/journal.pone.0285099 (PMC10146519; doi:10.1371/journal.pone.0285099)
Supplement: S2 Table — Panel A shows the results for virgin males from the major set of lines, Panel B shows the results for mixed males from the major set and Panel C the results for mixed males from the minor set of lines. As per Table 2, peaks are bolded, underlined or neither if they are classified as major, intermediate or minor in abundance. Their sex bias (also taken from Table 2) in samples from single/mixed sex cohorts is indicated as follows: Msp = male specific, Mse = male selective, Fse = female specific, Fse = female selective, nd = not detected and ~ = not sex biased, and virgin and mixed sex mating history categories are indicated before and after the slash, respectively. FDR-corrected significance values for F statistics are indicated with asterisks (* p < 0.05, ** p < 0.01, *** p< 0.001). The OrderNorm transformation used to ensure normality and homoscedacity also scaled the values to zero mean and unit variance. Confidence limits are given in parentheses. Letter codes are used to show the results of post hoc pairwise contrasts between lines, where lines with the same letters are not significantly different from one another. Note that peaks 4.17 and 8.06 in Panel C do not appear in Table 2 because they did not meet the inclusion criterion (detected in > 50% of samples in at least one sex/mating status category in at least one line) for analysis in the major set of lines and therefore were not classified for abundance or sex bias. (DOCX) [file pone.0285099.s005.docx]

**S2 Table.** Emmeans for OrderNorm transformed peak areas, plus their 95% confidence limits, for peaks showing significant variation between lines.

| **MAJOR SET OF ISOFEMALE LINES** | | | | | | | | | | | | | | | | | | | | | | | | | | | | | | | | |
| --- | --- | --- | --- | --- | --- | --- | --- | --- | --- | --- | --- | --- | --- | --- | --- | --- | --- | --- | --- | --- | --- | --- | --- | --- | --- | --- | --- | --- | --- | --- | --- | --- |
| 1. **Virgin males** | | | | | | | | | | | | | | | | | | | | | | | | | | | | | | | | |
| **Peak** | **Specificity/**  **selectivity** | | **F** | | **AS09** | | | **AS19** | | | | **AS36** | | | **CT07** | | **CT38** | | | | **CT60** | | **SY13** | | | | **SY18** | | **SY53** | | **S06** | |
| 4.61  *n*-Propyl 2-  methylpropa-  noate | Msp/Msp | | 8.87*** | | 0  (-0.5,0.6) | | | 0.4  (0,0.9) | | | | **-2**  (-3.3,-0.8) | | | 0.4  (-0.5,1.3) | | **-1.4**  (-2.3,-0.5) | | | | **-0.9**  (-1.6,-0.3) | | -0.4  (-0.9,0) | | | | -0.4  (-0.9,0.1) | | 0.2  (-0.4,0.7) | | **1.6**  (1.1,2.1) | |
| 5.20 | Msp/Msp | | 8.38*** | | **0.3**  (-0.2,0.8) | | | -0.1  (-0.5,0.4) | | | | -0.5  (-1.7,0.7) | | | -1.1  (-1.9,-0.2) | | -1.1  (-1.9,-0.2) | | | | -0.4  (-1,0.2) | | **0.4**  (0,0.8) | | | | **-1**  (-1.4,-0.5) | | 0  (-0.5,0.5) | | **1.5**  (1,2) | |
| 5.41 | Msp/Msp | | 5.10** | | 0.3  (-0.4,0.9) | | | -0.1  (-0.7,0.4) | | | | -0.2  (-1.7,1.3) | | | 0.8  (-0.3,1.8) | | **-2**  (-3,-0.9) | | | | -1  (-1.7,-0.2) | | 0.3  (-0.3,0.8) | | | | -0.4  (-1,0.3) | | -0.3  (-1,0.3) | | **1.3**  (0.7,1.9) | |
| 5.99 | Msp/Msp | | 3.30* | | 0.2  (-0.6,0.9) | | | **-0.6**  (-1.2,0) | | | | -0.2  (-1.8,1.5) | | | 0.2  (-1,1.3) | | **-1.6**  (-2.8,-0.4) | | | | 0.1  (-0.7,0.9) | | 0.1  (-0.5,0.7) | | | | -0.1  (-0.8,0.6) | | **-0.4**  (-1.2,0.3) | | **1.4**  (0.7,2) | |
| 6.30 | Msp/Msp | | 4.97** | | 0.2  (-0.5,0.9) | | | 0  (-0.6,0.5) | | | | -0.2  (-1.7,1.4) | | | 0.7  (-0.4,1.7) | | **-2**  (-3,-0.9) | | | | -1  (-1.7,-0.2) | | 0.2  (-0.3,0.7) | | | | -0.3  (-0.9,0.3) | | -0.4  (-1.1,0.3) | | **1.3**  (0.7,2) | |
| 6.64 | Msp/Msp | | 5.19** | | 0.5  (-0.1,1.1) | | | 0.3  (-0.2,0.8) | | | | -0.5  (-1.9,0.9) | | | 0.6  (-0.4,1.6) | | **-1.2**  (-2.2,-0.2) | | | | **-0.7**  (-1.4,0) | | **-0.8**  (-1.3,-0.3) | | | | **-0.2**  (-0.8,0.4) | | 0.2  (-0.5,0.8) | | **1.3**  (0.7,1.9) | |
| 10.05 | Msp/nd | | 4.66 ** | | 0.2  (-0.4,0.8) | | | **-0.4**  (-0.8,0.1) | | | | -0.8  (-2.2,0.5) | | | -0.4  (-1.4,0.5) | | **-0.8**  (-1.8,0.1) | | | | **-0.4**  (-1.1,0.3) | | 0.6  (0.1,1.1) | | | | **-0.5**  (-1.1,0) | | -0. 1  (-0.7,0.6) | | **1.3**  (0.8,1.9) | |
| **10.85** | Fse/Fse | | 3.40* | | **-0.4**  (-1,0.3) | | | **-0.6**  (-1.1,-0.1) | | | | -0.2  (-1.7,1.3) | | | -0.4  (-1.4,0.7) | | -0.6  (-1.6,0.5) | | | | 0.1  (-0.7,0.8) | | 0  (-0.5,0.6) | | | | **1.3**  (0.7,1.9) | | 0.6  (-0.1,1.3) | | -0.1  (-0.7,0.5) | |
| **13.47**  ***N*-2-methyl-**  **butyl-2-methylpro-panamide** | Nb~/Nb~ | | 5.50** | | 0.2  (-0.4,0.9) | | | **-0.2**  (-0.7,0.4) | | | | -0.2  (-1.7,1.3) | | | 0.4  (-0.6,1.5) | | **-1.1**  (-2.2,-0.1) | | | | **-0.3**  (-1.1,0.4) | | **-0.7**  (-1.2,-0.2) | | | | 0.6  (0,1.2) | | **-0.8**  (-1.4,-0.1) | | **1.5**  (0.9,2.1) | |
| 16.18 | Msp/Msp | | 9.56*** | | 0.8  (0.2,1.3) | | | **0.9**  (0.4,1.3) | | | | -0.3  (-1.5,0.9) | | | 0  (-0.9,0.8) | | -**1.4**  (-2.3,-0.5) | | | | -0.4  (-1,0.2) | | -0.4  (-0.8,0.1) | | | | **-0.9**  (-1.4,-0.5) | | **-0.8**  (-1.3,-0.3) | | **1.1**  (0.6,1.6) | |
| 17.07 | Msp/Msp | | 3.18* | | 0.3  (-0.5,1) | | | -0.3  (-0.9,0.3) | | | | -1.1  (-2.8,0.6) | | | 0.7  (-0.5,1.9) | | **-1.8**  (-3,-0.6) | | | | -0.4  (-1.2,0.5) | | 0.2  (-0.4,0.8) | | | | -0.1  (-0.8,0.6) | | -0.4  (-1.2,0.3) | | **1.2**  (0.5,1.8) | |
| 17.37  Methyl  dodecanoate | Nb~/Nb~ | | 5.73** | | 0.3  (-0.4,0.9) | | | **-0.2**  (-0.7,0.3) | | | | -0.7  (-2.1,0.7) | | | -0.4  (-1.4,0.7) | | **-0.9**  (-1.9,0.1) | | | | **-0.7**  (-1.4,0) | | **-0.8**  (-1.3,-0.3) | | | | **0.1**  (-0.5,0.7) | | 0.5  (-0.1,1.1) | | **1.6**  (1,2.2) | |
| **17.69** | Fse/Fse | | 3.53* | | 0.2  (-0.5,1) | | | **-0.2**  (-0.8,0.4) | | | | -0.8  (-2.5,0.8) | | | 0.4  (-0.7,1.6) | | **-1**  (-2.1,0.2) | | | | **-0**.6  (-1.4,0.2) | | **-0.4**  (-1,0.1) | | | | 0.5  (-0.2,1.1) | | **-0.6**  (-1.3,0.2) | | **1.4**  (0.7,2) | |
| 20.35 | Msp/nd | | 3.22* | | **1.3**  (0.6,2) | | | **-0.2**  (-0.8,0.3) | | | | 0.8  (-0.6,2.3) | | | 0.4  (-0.7,1.4) | | -0.2  (-1.3,0.8) | | | | **-0.4**  (-1.2,0.3) | | 0.6  (0,1.1) | | | | **-0.4**  (-1,0.2) | | **-0.4**  (-1,0.3) | | **-0.3**  (-0.9,0.3) | |
| 20.50 | Nb~/Nb~ | | 3.54* | | 0.6  (-0.1,1.3) | | **-0.6**  (-1.2,-0.1) | | | | | -1.1  (-2.6,0.4) | | | 1.1  (0,2.2) | | -0.7  (-1.8,0.3) | | | | 0.4  (-0.3,1.2) | | -0.2  (-0.7,0.4) | | | | -0.1  (-0.7,0.5) | | -0.4  (-1.1,0.3) | | **1**  (0.4,1.7) | |
| 20.59 | Msp/Msp | | 3.80* | | **0.8**  (0.1,1.5) | | | -0.6  (-1.2,-0.1) | | | | -0.5  (-2.1,1.1) | | | **1.3**  (0.2,2.4) | | -0.9  (-2,0.2) | | | | -0.1  (-0.9,0.7) | | 0.2  (-0.4,0.7) | | | | 0.2  (-0.4,0.9) | | **-1**  (-1.7,-0.3) | | **0.7**  (0.1,1.4) | |
| 20.98 | Msp/Msp | | 4.83** | | 0.6  (-0.1,1.2) | | | **-0.6**  (-1.1,-0.1) | | | | **-1.2**  (-2.6,0.2) | | | 0.1  (-0.9,1.1) | | **-0.9**  (-2,0.1) | | | | **-0.3**  (-1,0.4) | | **-0.3**  (-0.8,0.2) | | | | **-0.1**  (-0.7,0.5) | | 0.3  (-0.3,0.9) | | **1.4**  (0.8,2) | |
| 1. **Mixed males** | | | | | | | | | | | | | | | | | | | | | | | | | | | | | | | | |
| 18.68 | Msp/Msp | | 26.71* | | **1.4**  (0.9,1.8) | | | | 0.9  (0.4,1.3) | | | |  | | **-1.71**  (-2.4,-1.) | | |  | | |  | | | **-0.9**  (-1.3,-0.4) | | | **-0.9**  (-1.4,-0.4) | | -0.1  (-0.8,0.5) | | **0.3**  (-0.1 , 0.7) | |
| 20.15 | Fsp/Msp | | 24.05* | | 0.7  (0.3,1.1) | | | | -0.1  (-0.6,0.4) | | | |  | | 0.4  (-0.2,1.) | | |  | | |  | | | -0.7  (-1.2,-0.3) | | | **1.5**  (1.0,2) | | 0.19  (-0.4,0.8) | | **-1**  (-1.4 , -0.7) | |
| **MINOR SET OF ISOFEMALE LINES** | | | | | | | | | | | | | | | | | | | | | | | | | | | | | | | |  |
| 1. **Mixed males** | | | | | | | | | | | | | | | | | | | | | | | | | | | | | | | |  |
| **Peak** | **Specificity/**  **selectivity** | **F** | | **BR05** | | **BR34** | | | | **BR39** | **BR50** | | | **MB09** | **MB46** | **MB50** | | | **MB64** | **NB02** | | **NB11** | | | **NB28** | **UT01** | | **UT03** | | **UT43** | **S06** |  |
| 4.17 | **-** | 2.72* | | -0.8  (-1.5, -0.1) | | -0.5  (-1.1,0) | | | | 0  (-1.1,1.1) | -0.4  (-0.8,0) | | | 0.6  (0.1,1) | 0.6  (0,1.2) | 0.1  (-1,1.2) | | | 0.4  (0,0.8) | 0.3  (-0.2,0.8) | | 0.3  (-0.6,1.2) | | | 0.5  (0,1) | -0.1  (-0.9,0.7) | | -0.1  (-0.7,0.6) | | 0.3  (-1.3,1.8) | -0.8  (-1.4,-0.2) a |  |
| 4.61  *n*-Propyl 2-  methylpropa-  noate | Msp/Msp | 3.01* | | 0.6  (-0.1,1.4) | | 0.3  (-0.3,0.9) | | | | -0.1  (-1.3,1.1) | -0.1  (-0.5,0.4) | | | -0.1  (-0.6,0.3) | -0.2  (-0.8,0.5) | -1.1  (-2.3,0.1) | | | 0  (-0.5,0.5) | -0.6  (-1.1,0) | | -0.2  (-1.2,0.8) | | | -0.1  (-0.6,0.5) | -0.2  (-1,0.6) | | -0.1  (-0.8,0.6) | | 0.7  (-1,2.3) | 1.9  (1.2,2.6) |  |
| 5.41 | Msp/Msp | 3.18* | | 0.3  (-0.4, 1.1) | | 0  (-0.6,0.6) | | | | 0.7  (-0.6,1.9) | -0.7  (-1.2,-0.3) | | | 0.4  (-0.2,0.9) | 0  (-0.6,0.7) | -0.9  (-2.1,0.3) | | | 0  (-0.5,0.4) | -0.2  (-0.8,0.3) | | 0.4  (-0.6,1.4) | | | -0.4  (-1,0.2) | 0  (-0.9,0.9) | | -0.1  (-0.8,0.6) | | 0.7  (-1.1,2.4) | 1.8  (1.1,2.5) |  |
| 6.06 | Nb~/Nb~ | 3.03* | | 0.2  (-0.6,1) | | 0.4  (-0.2,1) | | | | 1  (-0.2,2.2) | 0.4  (0,0.9) | | | -0.5  (-1.1,0) | -0.6  (-1.2,0.1) | -1  (-2.3,0.2) | | | -0.5  (-1,0) | 0  (-0.5, 0.6) | | -0.2  (-1.2,0.8) | | | -0.4  (-1,0.2) | 0.4  (-0.5,1.3) | | 0.1  (-0.6,0.8) | | 0.2  (-1.5,1.9) | 1.5  (0.8,2.2) |  |
| 6.21 | M~/Msp | 2.73* | | 0.3  (-0.5,1) | | 0.4  (-0.3,1) | | | | 1.2  (-0.1,2.4) | 0.4  (-0.1,0.8) | | | -0.6  (-1.1,-0.1) | -0.6  (-1.2,0.1) | -1  (-2.3,0.2) | | | -0.5  (-1,0) | 0.1  (-0.5, 0.6) | | -0.2  (-1.2,0.9) | | | -0.4  (-1,0.2) | 0.3  (-0.6,1.2) | | 0.4  (-0.4,1.1) | | 0.2  (-1.6,2) | 1.3  (0.5,2) |  |
| 6.30 | Msp/Msp | 3.05* | | 0.8  (0.1,1.5) | | -0.2  (-0.8, 0.3) | | | | -0.6  (-1.8,0.5) | -0.7  (-1.1,-0.3) | | | 0.5  (0,1) | 0.2  (-0.4,0.8) | -0.3  (-1.4,0.9) | | | 0.1  (-0.3,0.6) | -0.3  (-0.8, 0.2) | | 0.6  (-0.3,1.5) | | | 0  (-0.5,0.5) | -0.5  (-1.3,0.3) | | -0.1  (-0.7,0.6) | | 1.2  (-0.5,2.8) | 1.2  (0.5,1.8) |  |
| 8.06 | - | 2.94* | | 0.9  (0.1,1.6) | | -0.1  (-0.7,0.4) | | | | 0.4  (-0.8,1.5) | 0.5  (0.1,1) | | | -0.4  (-0.9,0) | -0.4  (-1,0.2) | -0.6  (-1.8,0.5) | | | -0.2  (-0.7,0.2) | -0.2  (-0.7, 0.3) | | -0.4  (-1.3,0.5) | | | -0.4  (-1,0.1) | 0.3  (-0.5,1.1) | | 0.4  (-0.3,1) | | 0.1  (-1.5,1.7) | 1.3  (0.6,1.9) |  |
| 10.41  *N*-(2-methylbutyl)  acetamide | Mse/Msp | 2.54* | | 1  (0.2,1.9) | | 0.3  (-0.3,1) | | | | -0.5  (-1.7,0.8) | -0.3  (-0.7,0.2) | | | 0.3  (-0.3,0.8) | 0.2  (-0.5,0.9) | -1.1  (-2.3,0.2) | | | -0.3  (-0.8,0.2) | -0.7  (-1.2,-0.1) | | 0.4  (-0.6,1.5) | | | -0.1  (-0.7,0.5) | -0.7  (-1.6,0.2) | | -0.1  (-0.8,0.7) | | 1  (-0.8,2.8) | 1.2  (0.5,1.9) |  |
| **10.85** | Fse/Fse | 3.18* | | -0.6  (-1.3,0.2) | | 1  (0.4,1.6) | | | | 1.1  (0,2.3) | 0.5  (0.1,0.9) | | | -0.2  (-0.7,0.3) | -0.4  (-1.1,0.2) | -0.6  (-1.8,0.5) | | | -0.1  (-0.5,0.4) | -0.7  (-1.2,-0.2) | | -0.1  (-1,0.9) | | | 0.7  (0.2,1.3) | -0.2  (-1,0.6) | | -0.3  (-0.9,0.4) | | -1.3  (-3,0.4) | 0  (-0.7,0.7) |  |
| 19.80 | Nb~/Nb~ | 2.61* | | -0.6  (-1.4,0.1) | | 0.3  (-0.3,0.8) | | | | 0.9  (-0.3,2) | 0.5  (0.1,0.9) | | | 0.2  (-0.3,0.7) | -0.3  (-0.9,0.3) | 0  (-1.1,1.2) | | | -0.7  (-1.1,-0.2) | 0.3  (-0.2, 0.8) | | -0.1  (-1,0.8) | | | 0.5  (0,1.1) | 0  (-0.8,0.9) | | 0.4  (-0.2,1.1) | | -0.9  (-2.5,0.7) | -0.8  (-1.4,-0.1) |  |
| 19.83 | Msp/Msp | 2.64* | | -0.2  (-1,0.6) | | -0.1  (-0.8,0.5) | | | | 0.5  (-0.8,1.7) | 0.7  (0.3,1.2) | | | 0.5  (0,1) | -0.3  (-1,0.3) | -0.8  (-2.1,0.4) | | | -0.4  (-0.9,0.1) | -0.3  (-0.9, 0.3) | | -0.2  (-1.2,0.8) | | | 0.3  (-0.3,0.9) | -0.1  (-1,0.8) | | 0.7  (0,1.4) | | -0.8 (-2.6,1) | -1.2 (-1.9,-0.5) |  |
