# Supplementary material for: Genetic variation for rectal gland volatiles among recently collected isofemale lines and a domesticated strain of Queensland fruit fly, Bactrocera tryoni (Diptera: Tephritidae)
Source: PLoS One. 2023 Apr 28;18(4):e0285099. doi: 10.1371/journal.pone.0285099 (PMC10146519; doi:10.1371/journal.pone.0285099)
Supplement: S3 Table — KIs given in that study and abbreviations used in S1 and S3 Figs are also shown. Four additional compounds tentatively identified by GC-MS in the current study were 2-methyl 3-hexanol, n-butylcyclopentane and two isomers of borneol (2M3H, NBCP, Bor1 and Bor2, respectively). (DOCX) [file pone.0285099.s006.docx]

**S3 Table.** Thirty seven previously identified rectal gland GC-MS peaks in Castro-Vargas et al.^⁕^

| **Compound** | **Abbreviation** | **KI obs** |
| --- | --- | --- |
| Ethyl propanoate | EP | 702 |
| Ethyl 2-methylpropanoate | E2MP | 752 |
| (*D*,*L*)- 2,3-Butanediol | 23But | 786 |
| (*meso*)- 2,3-Butanediol | 23But(meso) | 789 |
| Ethyl 2-methylbutanoate | E2MB | 843 |
| *n*-Propyl 2-methylpropanoate | P2MP | 857 |
| 4-Heptanone | 4Hep | 867 |
| *x*-Octenal isomer1 | Oct-01 | 902 |
| Ethyl 2-methylpentanoate | E2MPen | 933 |
| *x*-Octenal isomer 2 | Oct-02 | 939 |
| *n*-Octen-1-ol | Oct1ol | 1019 |
| 2-Ethyl-1-hexanol | 2E1H | 1026 |
| N-(2-Methylpropyl)propanamide | 2MPP | 1094 |
| *N*-(2-Methylbutyl)acetamide | Am1 | 1132 |
| *N*-(3-Methylbutyl)acetamide | Am2 | 1142 |
| (*E*,*E*)-2,8-Dimethyl-1,7-dioxaspiro[5.5]undecane | 28DDU | 1159 |
| 2-Bornanone | 2Bor | 1171 |
| Diethyl succinate | DS | 1182 |
| *N*-(2-Methylbutyl)propanamide | Am3 | 1201 |
| *N*-(3-Methylbutyl)propanamide | Am4 | 1212 |
| *N*-(2-Methylbutyl)-2-methylpropanamide | Am5 | 1235 |
| *N*-(3-Methylbutyl)-2-methylpropanamide | Am6 | 1239 |
| Methyl dodecanoate | MD | 1525 |
| Ethyl (*Z*)-9-dodecenoate | E9D | 1583 |
| Ethyl dodecanoate | ED | 1592 |
| *n*-Propyl dodecanoate | PD | 1675 |
| Methyl (Z)-9-tetradecenoate | M9T | 1716 |
| Methyl tetradecanoate | MT | 1723 |
| Ethyl (*E*)-9-tetradecenoate | E9T | 1783 |
| Ethyl tetradecanoate | ET | 1788 |
| Ethyl 12-methyltetradecanoate | E12MT | 1868 |
| *n*-Propyl tetradecanoate | PT | 1889 |
| Methyl (*Z*)-9-hexadecenoate | M9Hex | 1911 |
| Methyl hexadecanoate | MHex | 1928 |
| Ethyl (*Z*)-9-hexadecenoate | E9Hex | 1981 |
| Ethyl hexadecanoate | EHex | 1989 |
| Ethyl (*E*)-9-octadecenoate | E9Oct | 2188 |

*Castro-Vargas C, Pandey G, Yeap HL, Lacey MJ, Lee SF, Park SJ, et al. Diversity and sex differences in rectal gland volatiles of Queensland fruit fly, *Bactrocera tryoni* (Diptera: Tephritidae). PLoS One. 2022;17: e0273210. doi:10.1371/journal.pone.0273210
