# Supplementary material for: Genetic variation for rectal gland volatiles among recently collected isofemale lines and a domesticated strain of Queensland fruit fly, Bactrocera tryoni (Diptera: Tephritidae)
Source: PLoS One. 2023 Apr 28;18(4):e0285099. doi: 10.1371/journal.pone.0285099 (PMC10146519; doi:10.1371/journal.pone.0285099)
Supplement: S4 Table — For each of the two technologies, data are given for KI, maximum abundance (“Abund.”, i.e., relative abundance in the sex/mating history where it was highest; maj, int and min = major, intermediate and minor, respectively), sex-dependent abundances in virgin and mixed samples (“Sex differences”), and, where known, compound names. Rt values and significant line variation (“sig”) are also indicated for the GC-FID peaks. Peaks are listed in ascending KI order, with the data for calibrator peaks with the two technologies on the same line. Also shown on the same line are 27 pairs of peaks whose imputed GC-FID KI and observed GC-MS KI were no more than four units apart (Best match) and clearly more similar to each other than the immediately adjacent peaks (Next before and Next after). These 27 pairs were then further filtered in respect of their sex-dependent abundances in virgin and mixed samples (before or after the comma respectively, where M or F = present in all male and female samples, respectively, m or f = present in at least one but not all of the respective samples, nd = not detected, na = not assayed, and > or < represent differences of at least 10 fold in the mean peak areas for the two sexes). Compatibility was rated high (YY) for 15 pairs of peaks whose sex-specific expression in each type of sample was the same with both technologies (whether present in all replicates of each type of sample or not, for peaks 5.20, 6.64, 9.58, 10.05, 11.46, 11.70, 11.93, 11.97, 13.95, 14.37, 18.42) or not assayed with one technology (minor data set peaks 7.08, 8.06, 11.16, 11.50). Compatibility was considered acceptable (Y) for five other pairs showing a presence/absence difference in which the presence involved an m or f in a peak whose maximum abundance was still only minor (peaks 6.21, 13.88), or the presence still showed a greater than ten fold bias towards the sex in question (peaks 6.30, 17.07, 17.17). The remaining seven of the 27 KI-matched pairs were considered inco [file pone.0285099.s007.docx]

**S4 Table.** Alignment of GC-FID and GC-MS profiles in S06 males.

| GC-FID peaks | | | | | KI matches | | | GC-MS peaks | | | | Compatibility |
| --- | --- | --- | --- | --- | --- | --- | --- | --- | --- | --- | --- | --- |
| Names & line differences | Rts | KI(imp) | Abund. | Sex differences | Best match | Next before | Next after | Names | KI(obs) | Abund. | Sex differences |  |
| E2MB | 4.23 | 839 | int | Mf,MF | -4 | -7 | 14 | E2MB | 843 | int | M,M | N |
| P2MP (sig) | 4.61 | 857 | int | M,M |  |  |  |  |  |  |  |  |
| 4Hep | 4.82 | 867 | min | m,M | calibrator |  |  | 4Hep | 867 | min | m,M |  |
|  | 4.96 | 874 | min | M,M |  |  |  |  |  |  |  |  |
| 3MHex (sig) | 5.2 | 885 | int | M,M | 2 | -11 | 12 | 3MHex | 883 | int | M,M | YY |
| (sig) | 5.41 | 895 | int | M,M |  |  |  |  |  |  |  |  |
| Oct-01 |  |  |  |  |  |  |  | Oct-01 | 902 | int | M,M |  |
| (sig) | 5.99 | 922 | int | M,M |  |  |  |  |  |  |  |  |
|  | 6.02 | 924 | min | na,Mna |  |  |  |  |  |  |  |  |
| (sig) | 6.06 | 926 | int | MF,MF |  |  |  |  |  |  |  |  |
| E2MPen (sig) | 6.21 | 933 | min | Mf,Mf | 0 | -7 | 4 | E2MPen | 933 | min | M,M | Y |
| Oct-02 (sig) | 6.3 | 937 | min | M,M | -2 | -6 | 9 | Oct-02 | 939 | int | M>F,m>f | Y |
|  | 6.53 | 948 | min | na,Mna |  |  |  |  |  |  |  |  |
| BP | 6.64 | 953 | int | M,M | -2 | -7 | 12 | BP | 955 | min | M,M | YY |
|  | 6.94 | 967 | min | m,m |  |  |  |  |  |  |  |  |
| Phe | 7.08 | 974 | min | na,Mna | 2 | -5 | 28 | Phe | 972 | min | MF,M>f | YY |
| 2E4MP |  |  |  |  |  |  |  | 2E4MP | 981 | min | mf,mf |  |
|  |  |  |  |  |  |  |  |  | 992 | min | M,M |  |
|  | 7.66 | 1002 | int | M>f,MF |  |  |  |  |  |  |  |  |
|  | 7.79 | 1008 | min | nd,m |  |  |  |  |  |  |  |  |
| Oct1ol (sig) | 8.06 | 1021 | min | na,Mna | 2 | -11 | 17 | Oct1ol | 1019 | min | MF,MF | YY |
| 2E1H |  |  |  |  |  |  |  | 2E1H | 1026 | maj | MF,MF |  |
|  |  |  |  |  |  |  |  |  | 1028 | min | mf,MF |  |
|  | 8.38 | 1036 | int | Mf,MF |  |  |  |  |  |  |  |  |
|  | 8.53 | 1043 | min | m,M |  |  |  |  |  |  |  |  |
|  | 8.73 | 1052 | min | nd,m |  |  |  |  |  |  |  |  |
|  | 9.43 | 1086 | min | M,M |  |  |  |  |  |  |  |  |
| MPP | 9.58 | 1093 | int | M,M | -1 | -10 | 6 | MPP | 1094 | int | M,M | YY |
|  | 9.74 | 1100 | min | na,Mna |  |  |  |  |  |  |  |  |
|  | 9.79 | 1103 | min | m,m |  |  |  |  |  |  |  |  |
|  | 9.98 | 1112 | int | m,M |  |  |  |  |  |  |  |  |
| (sig) | 10.05 | 1115 | min | M,m | 0 | -3 | 17 |  | 1115 | min | m,M | YY |
|  |  |  |  |  |  |  |  |  | 1123 | min | m,M |  |
|  |  |  |  |  |  |  |  |  | 1127 | maj | m,m |  |
| Am1 | 10.41 | 1132 | int | M,M | calibrator |  |  | Am1 | 1132 | int | MF,M>F |  |
| Am2 | 10.59 | 1142 | maj | M>F,M>F | calibrator |  |  | Am2 | 1142 | maj | MF,M>F |  |
|  | 10.61 | 1143 | int | na,Mna |  |  |  |  |  |  |  |  |
| (sig) | 10.85 | 1151 | maj | m<F,m<F |  |  |  |  |  |  |  |  |
| 28DDU | 11.04 | 1157 | int | m,M | -2 | -8 | 2 | 28DDU | 1159 | maj | M<F,M<F | N |
|  | 11.16 | 1161 | min | na,Mna | -3 | -7 | 3 |  | 1164 | int | M,M | YY |
| 2Bor | 11.46 | 1171 | int | M,M | 0 | -10 | 1 | 2Bor | 1171 | min | M,nd | YY |
|  | 11.5 | 1172 | min | na,Mna | -1 | -2 | 6 |  | 1173 | min | m,nd | YY |
|  |  |  |  |  |  |  |  |  | 1175 | int | MF,Mf |  |
| B01 | 11.7 | 1179 | min | nd,m | 0 | -7 | 3 | B01 | 1179 | min | M,M | YY |
| DS | 11.79 | 1182 | min | nd,m | calibrator |  |  | DS | 1182 | min | M,M |  |
| B02 | 11.93 | 1185 | min | M,M | -2 | -5 | -1 | B02 | 1187 | min | M,M | YY |
|  | 11.97 | 1186 | min | m,M | -2 | -3 | 7 |  | 1188 | int | M,M | YY |
|  | 12.27 | 1193 | min | M,M |  |  |  |  | 1189 | int | m,m |  |
|  | 12.45 | 1197 | min | nd,M |  |  |  |  |  |  |  |  |
|  | 12.56 | 1199 | min | na,Mna |  |  |  |  |  |  |  |  |
| Am3 | 12.63 | 1201 | maj | mF,M | calibrator |  |  | Am3 | 1201 | maj | M>F,M>F |  |
| Am4 | 12.96 | 1212 | maj | MF,MF | calibrator |  |  | Am4 | 1212 | maj | MF,M>F |  |
|  |  |  |  |  |  |  |  |  | 1216 | int | mF,MF |  |
|  |  |  |  |  |  |  |  |  | 1219 | int | m<F,M<F |  |
|  | 13.33 | 1227 | maj | m,nd |  |  |  |  |  |  |  |  |
|  | 13.34 | 1228 | int | nd,m |  |  |  |  |  |  |  |  |
| Am5 (sig) | 13.47 | 1235 | maj | MF,MF | calibrator |  |  | Am5 | 1235 | maj | M>F,M>F |  |
| Am6 | 13.59 | 1239 | maj | M,M | calibrator |  |  | Am6 | 1239 | maj | MF,MF |  |
| 2E8MDU |  |  |  |  |  |  |  | 2E8MDU | 1244 | min | M<F,MF |  |
|  | 13.79 | 1248 | min | m,M |  |  |  |  |  |  |  |  |
|  | 13.82 | 1249 | min | na,Mna |  |  |  |  |  |  |  |  |
|  | 13.88 | 1252 | min | nd,M | -1 | -4 | 1 |  | 1253 | min | MF,Mf | Y |
|  | 13.95 | 1254 | int | M,m | -2 | -4 | 11 |  | 1256 | int | M,M | YY |
|  | 14.37 | 1267 | int | mF,MF | 0 | -13 | 4 |  | 1267 | min | MF,MF | YY |
|  | 14.86 | 1279 | int | Mf,MF |  |  |  |  |  |  |  |  |
|  | 15.07 | 1284 | min | m,nd |  |  |  |  |  |  |  |  |
|  | 15.22 | 1289 | min | M,M |  |  |  |  |  |  |  |  |
|  | 15.38 | 1295 | min | M,M | -1 | -7 | 2 |  | 1296 | min | f,MF | N |
|  | 15.45 | 1298 | min | m,m |  |  |  |  |  |  |  |  |
|  | 15.51 | 1300 | min | mf,mf |  |  |  |  |  |  |  |  |
|  | 15.59 | 1304 | min | MF,Mf | -1 | -5 | 3 |  | 1303 | min | M,M | N |
|  | 15.67 | 1308 | min | m.M |  |  |  |  |  |  |  |  |
|  | 15.77 | 1314 | int | MF,MF |  |  |  |  |  |  |  |  |
|  | 15.87 | 1320 | min | M,MF |  |  |  |  |  |  |  |  |
|  | 15.98 | 1328 | min | m,M |  |  |  |  |  |  |  |  |
|  | 15.99 | 1329 | int | m,m |  |  |  |  |  |  |  |  |
|  | 16.11 | 1338 | maj | M>F,M>F |  |  |  |  |  |  |  |  |
|  | 16.14 | 1341 | min | f,MF |  |  |  |  |  |  |  |  |
| (sig) | 16.18 | 1344 | min | M,M |  |  |  |  |  |  |  |  |
|  | 16.32 | 1358 | int | MF,MF | 1 | -13 | 15 |  | 1357 | maj | F,MF | N |
|  | 16.45 | 1372 | int | MF,MF |  |  |  |  |  |  |  |  |
|  | 16.5 | 1377 | min | f,MF |  |  |  |  |  |  |  |  |
|  | 16.52 | 1380 | min | M,M |  |  |  |  |  |  |  |  |
|  | 16.59 | 1389 | min | M,M |  |  |  |  |  |  |  |  |
|  | 16.66 | 1398 | min | m,M |  |  |  |  |  |  |  |  |
|  | 16.7 | 1403 | min | m,M |  |  |  |  |  |  |  |  |
|  | 16.73 | 1408 | min | m,M |  |  |  |  |  |  |  |  |
|  | 16.78 | 1415 | min | m,M |  |  |  |  |  |  |  |  |
|  | 16.85 | 1426 | min | m,M |  |  |  |  |  |  |  |  |
|  | 16.89 | 1432 | min | m,M |  |  |  |  |  |  |  |  |
|  | 16.94 | 1440 | int | M,M | -2 | -10 | 10 |  | 1442 | int | MF,M | N |
|  | 17.01 | 1452 | min | M,M | -2 | -14 | 9 |  | 1454 | min | MF,M | N |
| (sig) | 17.07 | 1463 | min | M,M | -1 | -12 | 14 |  | 1464 | int | M,M>F | Y |
|  |  |  |  |  |  |  |  |  | 1468 | min | m,M |  |
|  | 17.15 | 1478 | min | m,M |  |  |  |  |  |  |  |  |
|  | 17.17 | 1482 | min | m,M | -2 | -6 | 6 |  | 1484 | int | M>F,M | Y |
|  | 17.21 | 1490 | int | MF,MF |  |  |  |  |  |  |  |  |
|  | 17.26 | 1501 | min | m,M |  |  |  |  |  |  |  |  |
|  | 17.32 | 1514 | int | Mf,Mf |  |  |  |  |  |  |  |  |
| MD (sig) | 17.37 | 1525 | int | MF,MF |  |  |  |  |  |  |  |  |
|  | 17.41 | 1535 | min | m,m |  |  |  |  |  |  |  |  |
|  | 17.45 | 1546 | int | MF,MF |  |  |  |  |  |  |  |  |
|  | 17.47 | 1551 | min | na,Mna |  |  |  |  |  |  |  |  |
|  | 17.52 | 1564 | min | M,Mf |  |  |  |  |  |  |  |  |
|  | 17.55 | 1571 | maj | m<F,M<F |  |  |  |  |  |  |  |  |
| E9D |  |  |  |  |  |  |  | E9D | 1583 | int | MF,MF |  |
| ED | 17.63 | 1592 | int | M,Mf |  |  |  |  |  |  |  |  |
|  | 17.64 | 1594 | Int | m,m |  |  |  |  |  |  |  |  |
|  | 17.69 | 1606 | maj | M<F,M<F |  |  |  |  |  |  |  |  |
|  | 17.7 | 1608 | min | na,Mna |  |  |  |  |  |  |  |  |
|  | 17.72 | 1612 | min | m,nd |  |  |  |  |  |  |  |  |
|  | 17.75 | 1619 | int | m,M |  |  |  |  |  |  |  |  |
|  | 17.84 | 1639 | Int | M,M |  |  |  |  |  |  |  |  |
|  | 17.92 | 1658 | int | m>f, m |  |  |  |  |  |  |  |  |
|  | 17.96 | 1667 | int | mF,mF |  |  |  |  |  |  |  |  |
|  | 18.01 | 1678 | int | M,M |  |  |  |  |  |  |  |  |
|  | 18.02 | 1680 | int | nd,m |  |  |  |  |  |  |  |  |
|  | 18.1 | 1698 | int | MF,MF |  |  |  |  |  |  |  |  |
|  | 18.12 | 1703 | int | mf,M |  |  |  |  |  |  |  |  |
|  | 18.17 | 1714 | int | MF,MF |  |  |  |  |  |  |  |  |
| MT | 18.21 | 1723 | int | mf,MF |  |  |  |  |  |  |  |  |
|  | 18.27 | 1740 | int | MF,MF |  |  |  |  |  |  |  |  |
|  | 18.32 | 1754 | int | MF,MF |  |  |  |  |  |  |  |  |
|  |  |  |  |  |  |  |  | EE9T | 1760 | maj | M<F,M<F |  |
|  | 18.37 | 1769 | int | mF,mF |  |  |  |  |  |  |  |  |
|  | 18.39 | 1774 | int | mF,F |  |  |  |  |  |  |  |  |
| E9T | 18.42 | 1783 | maj | mF,MF | 0 | -9 | 12 |  | 1783 | maj | m<F,M<F | YY |
